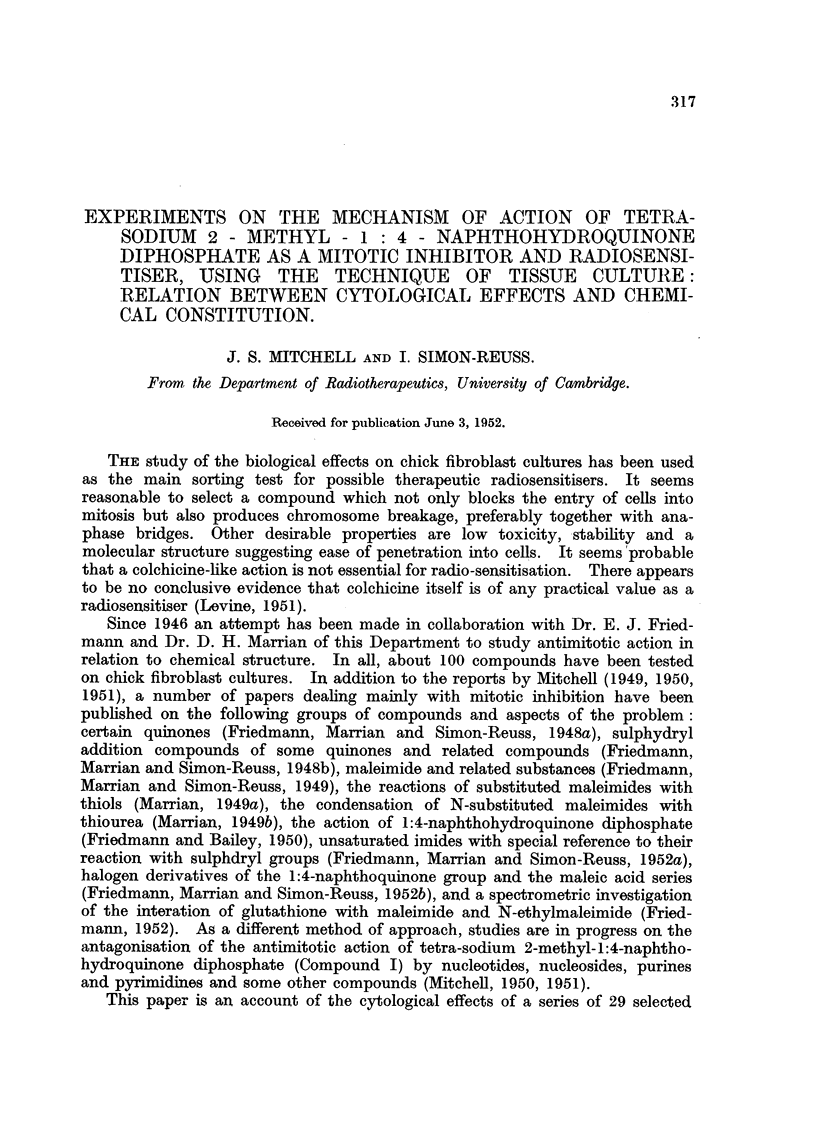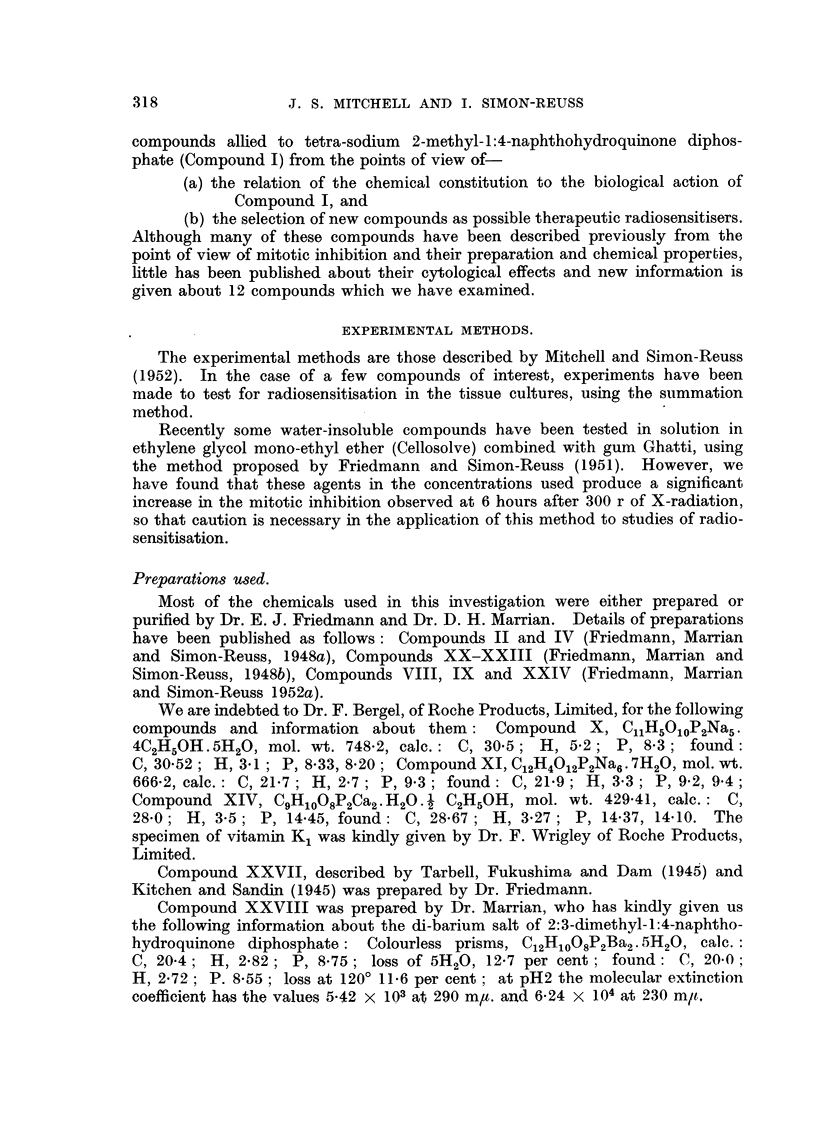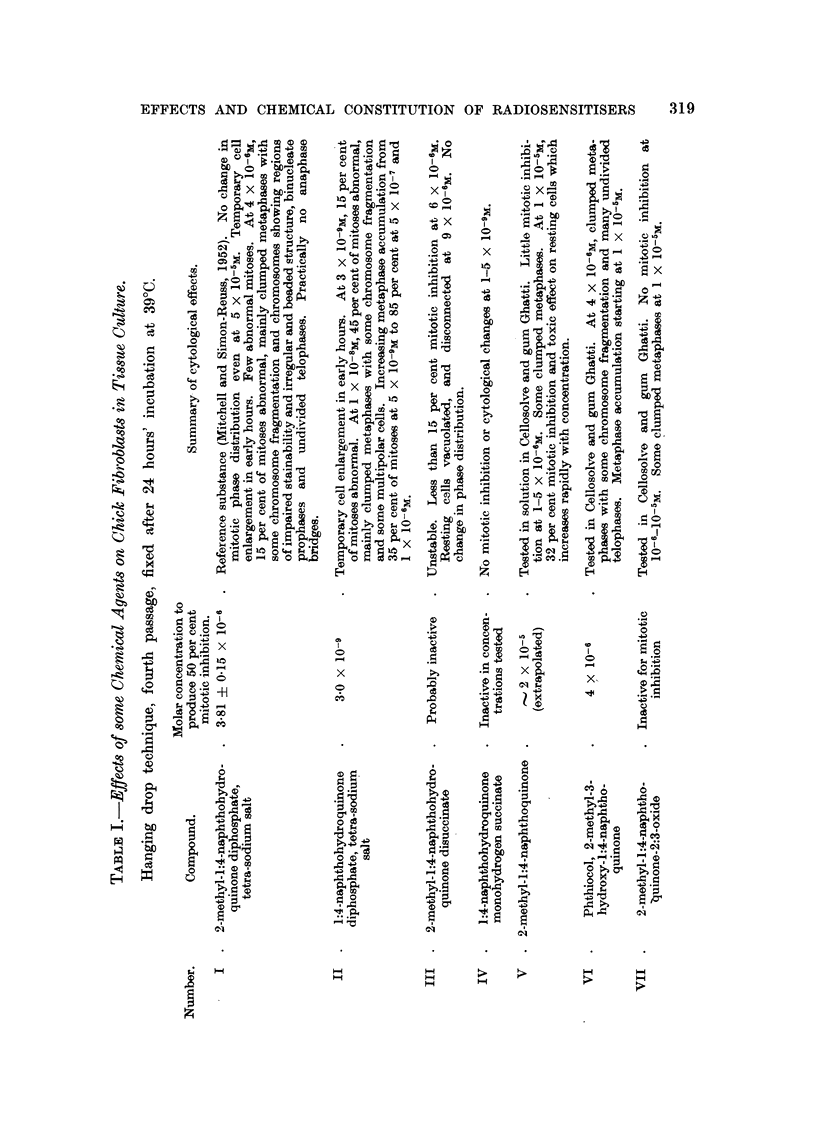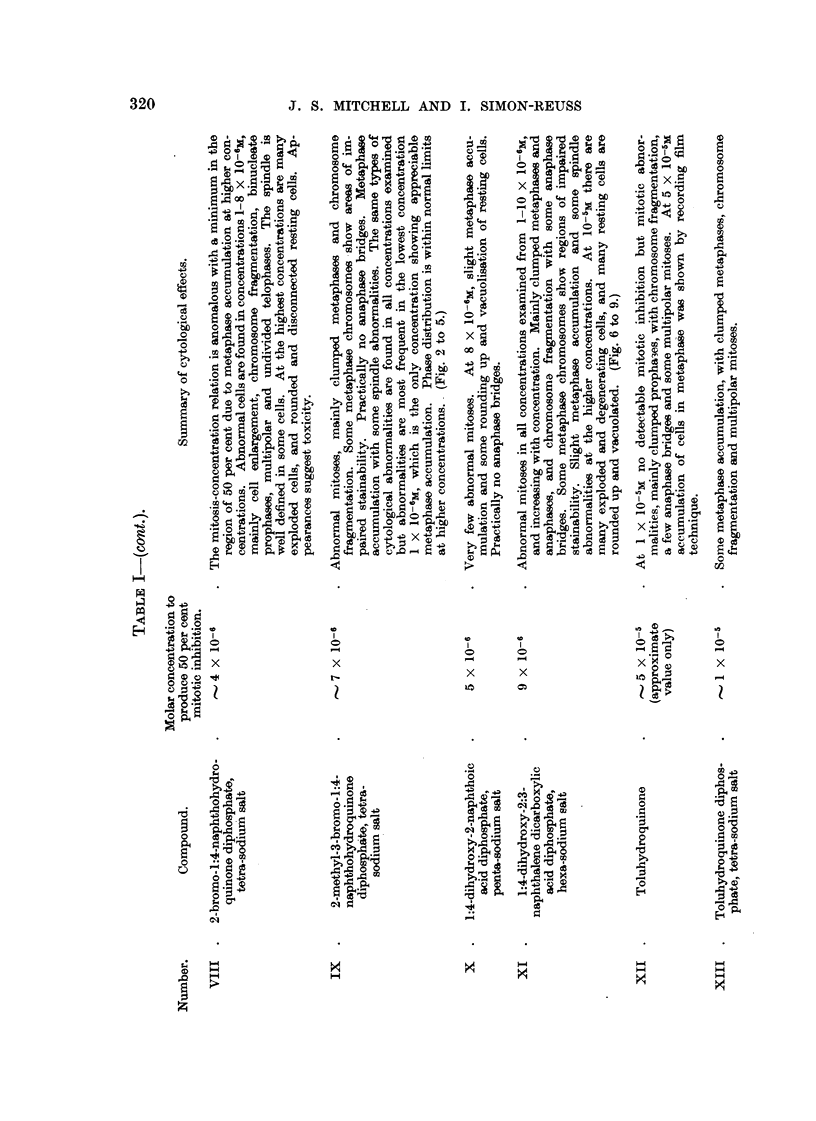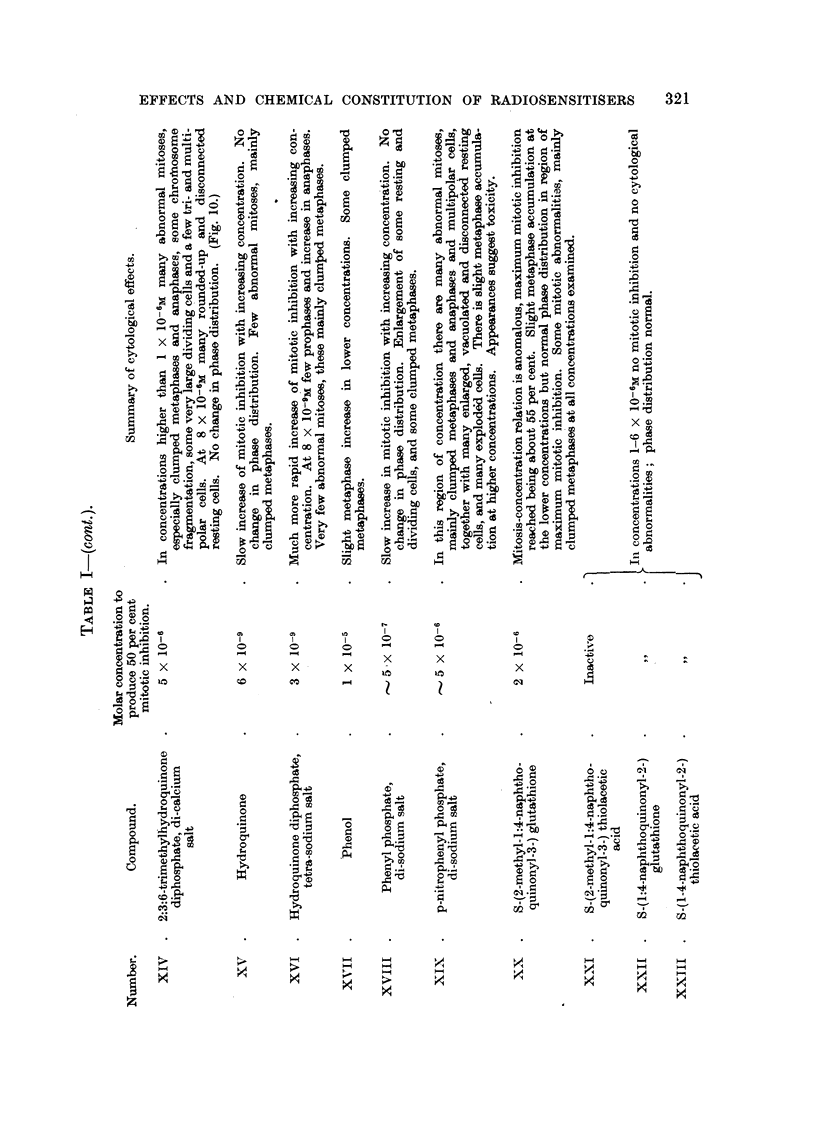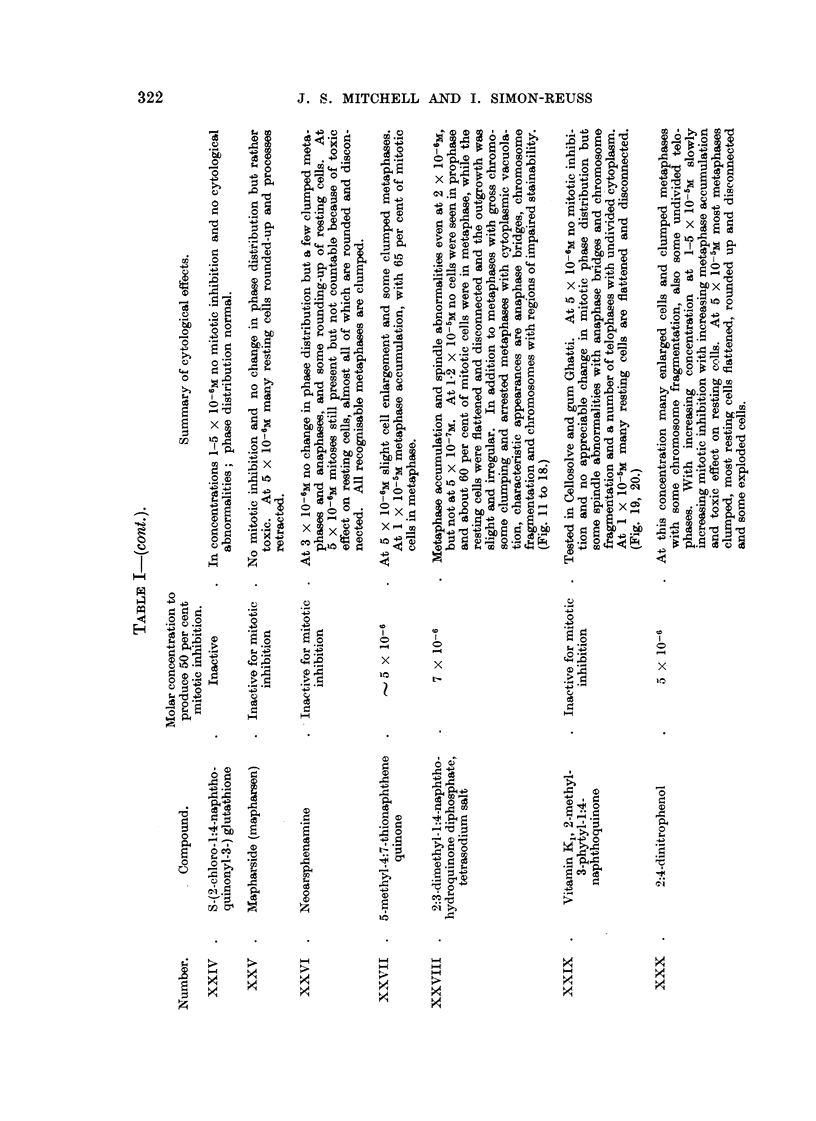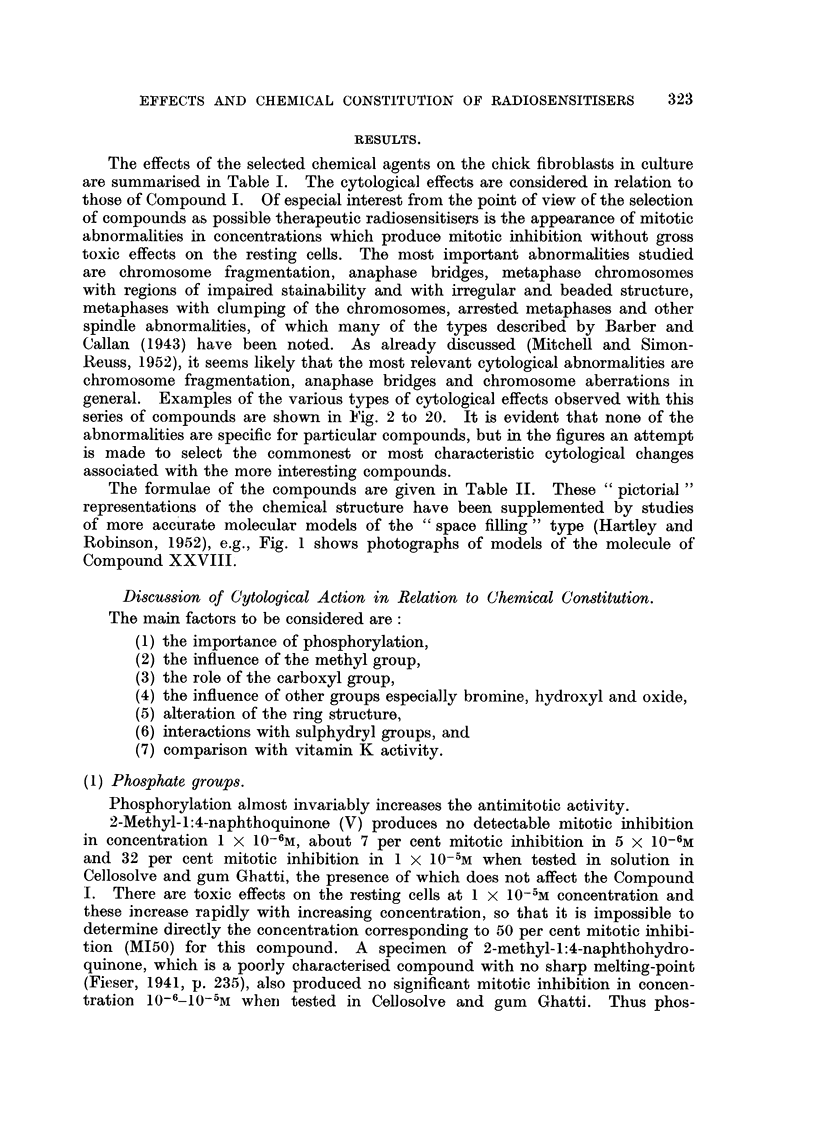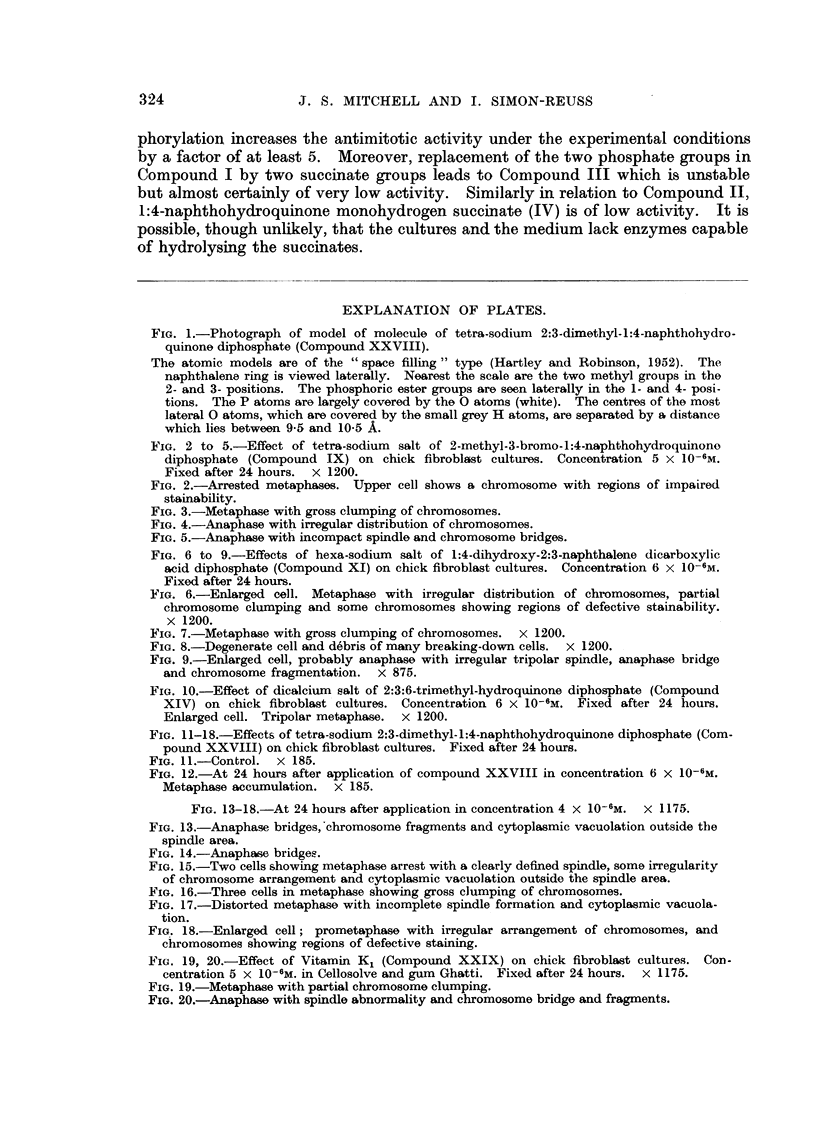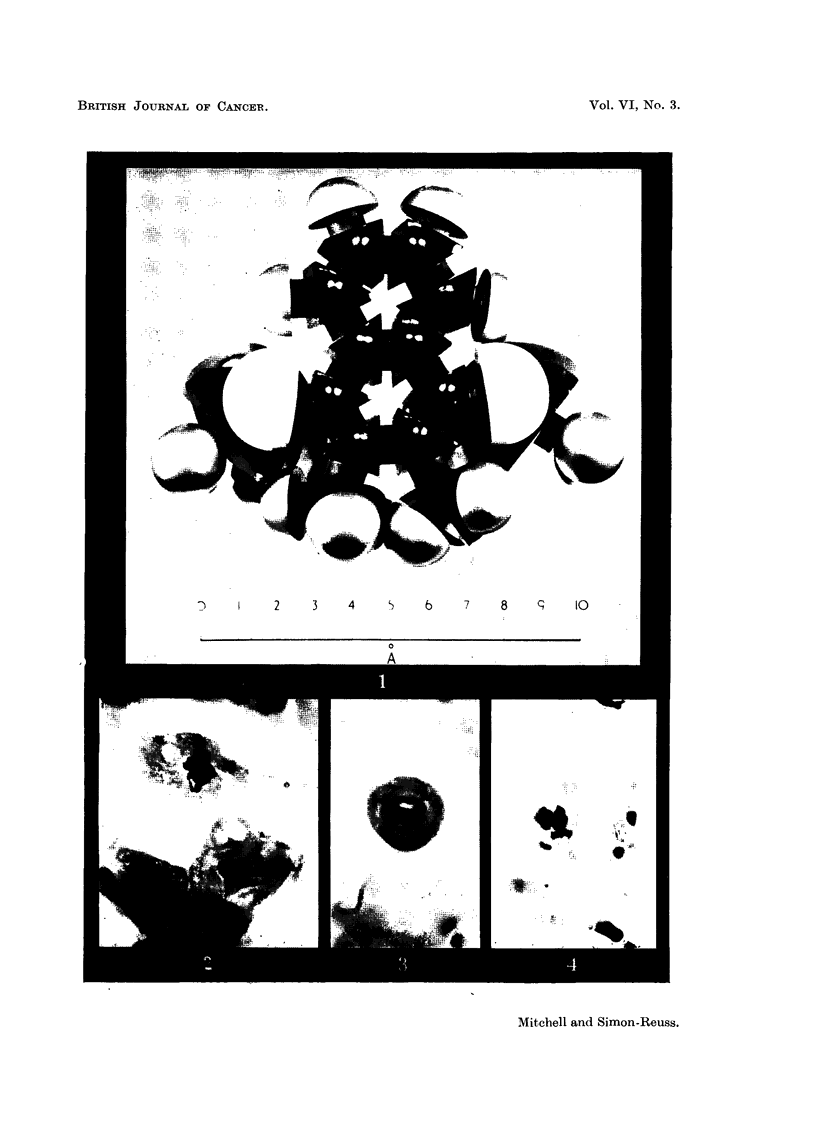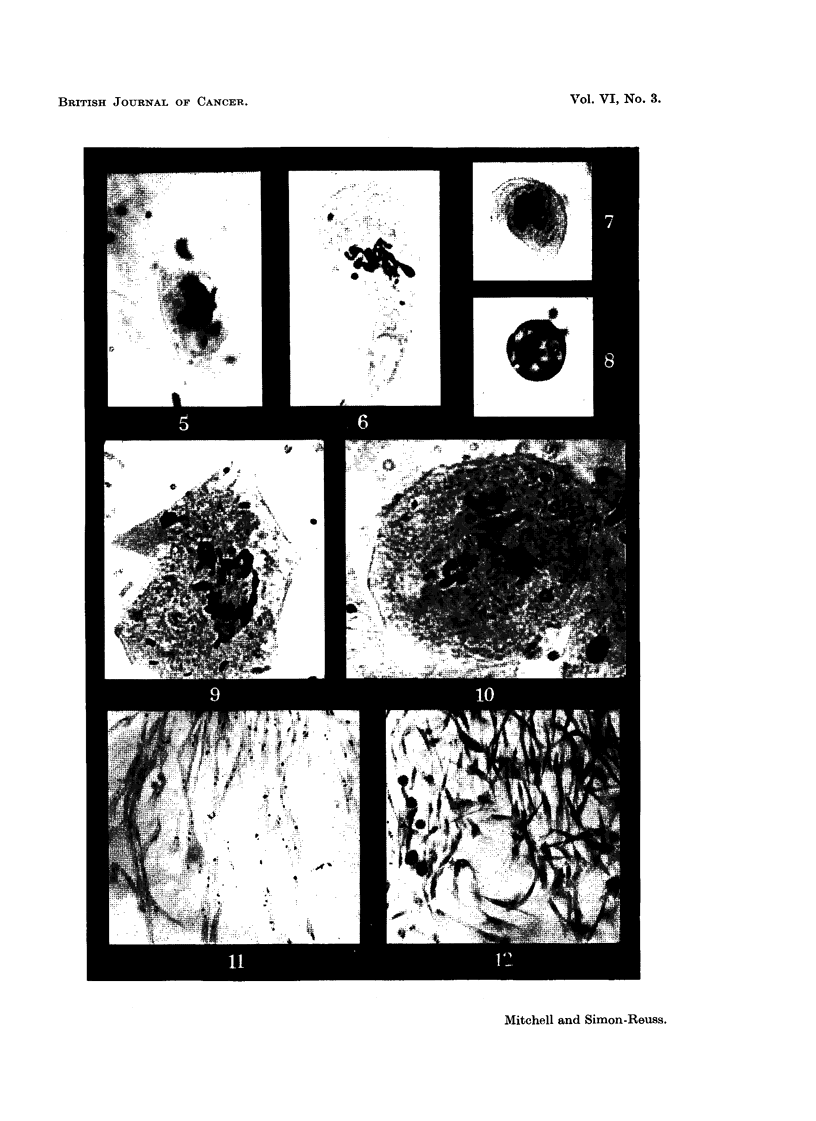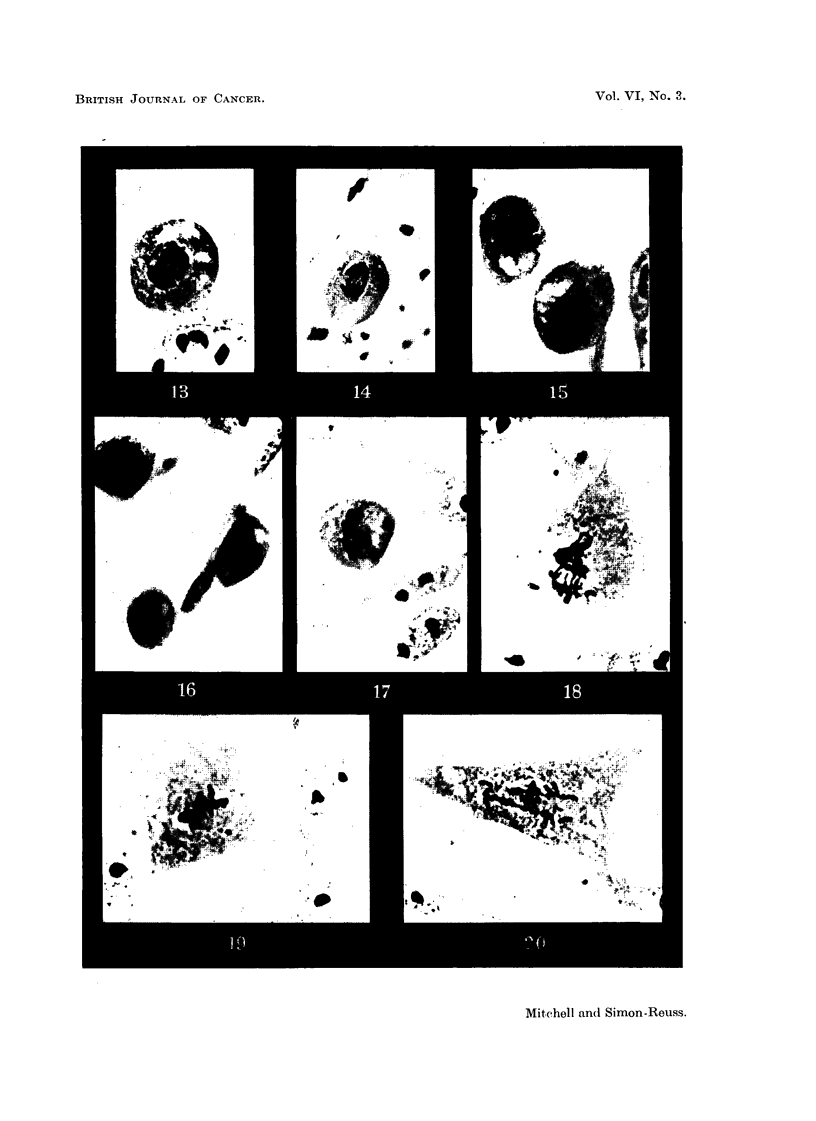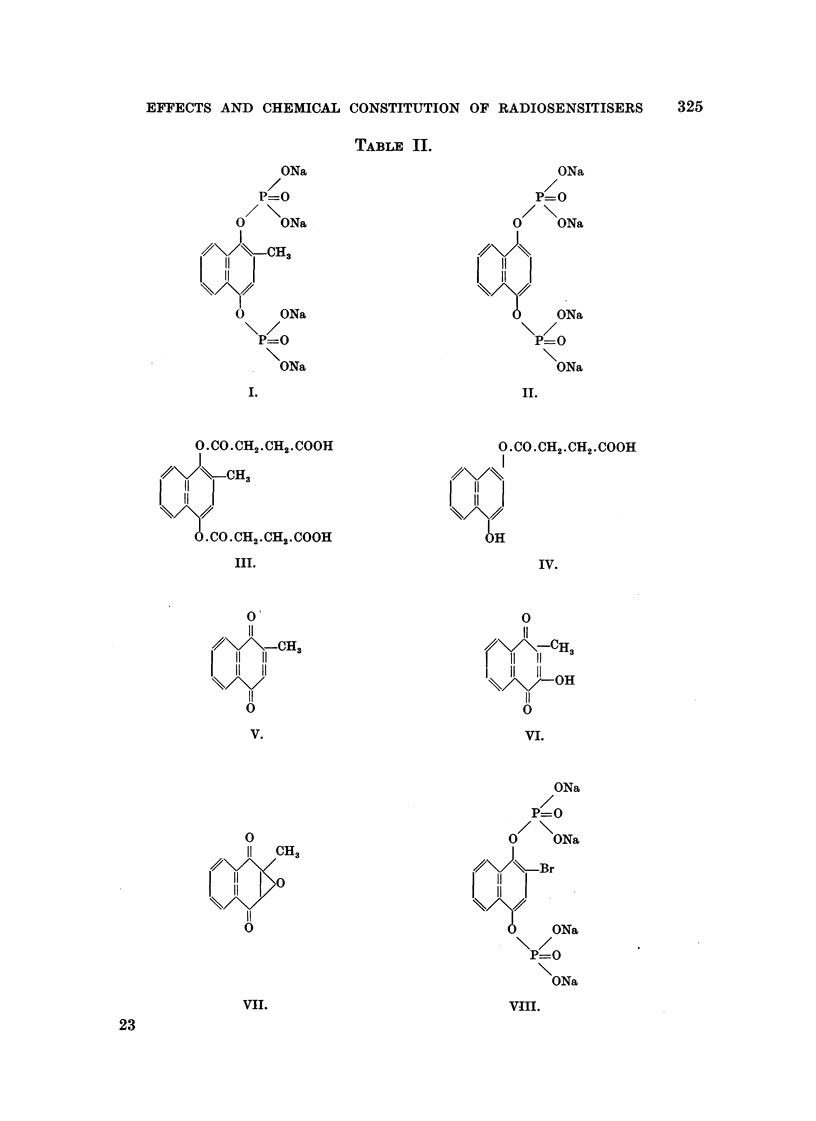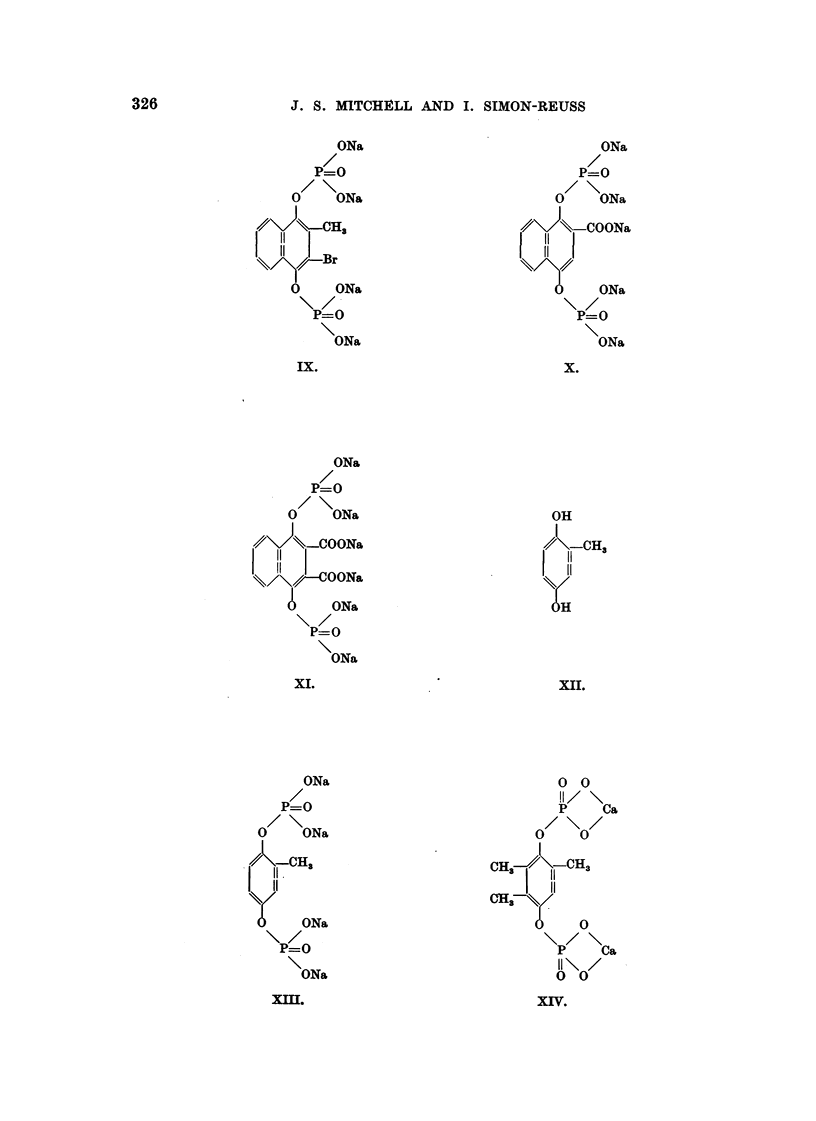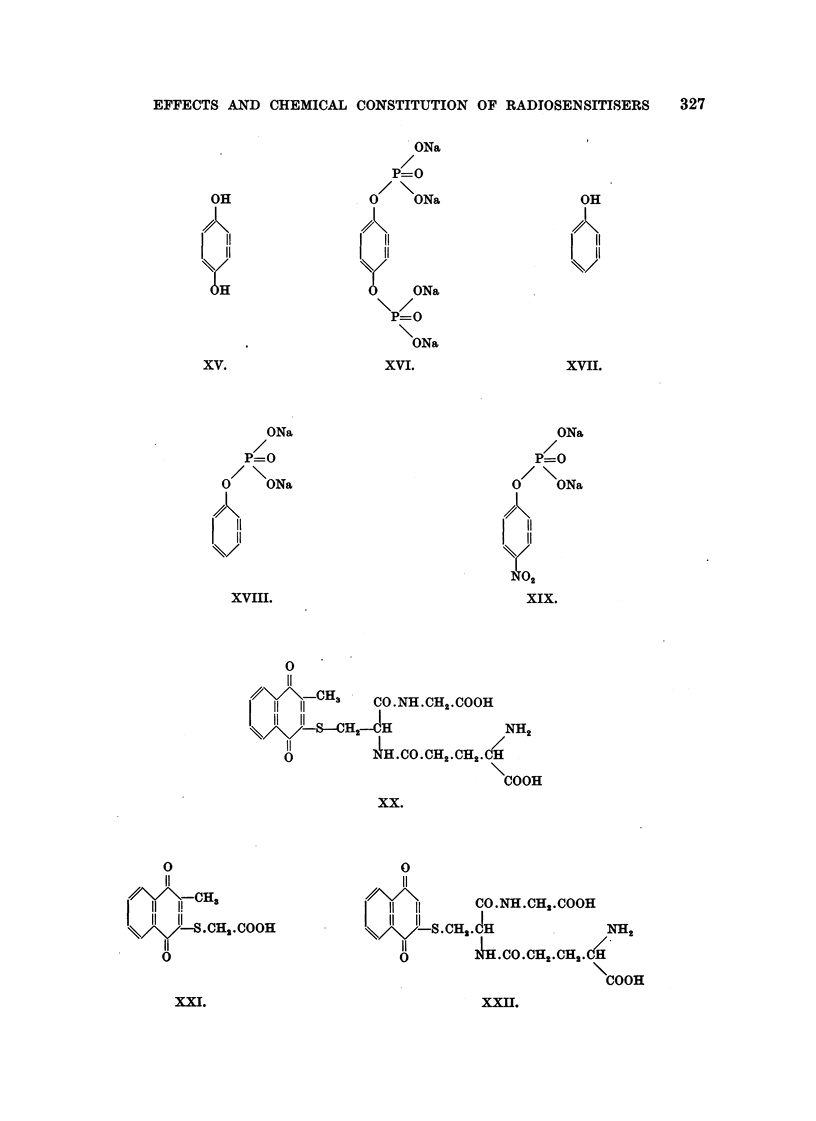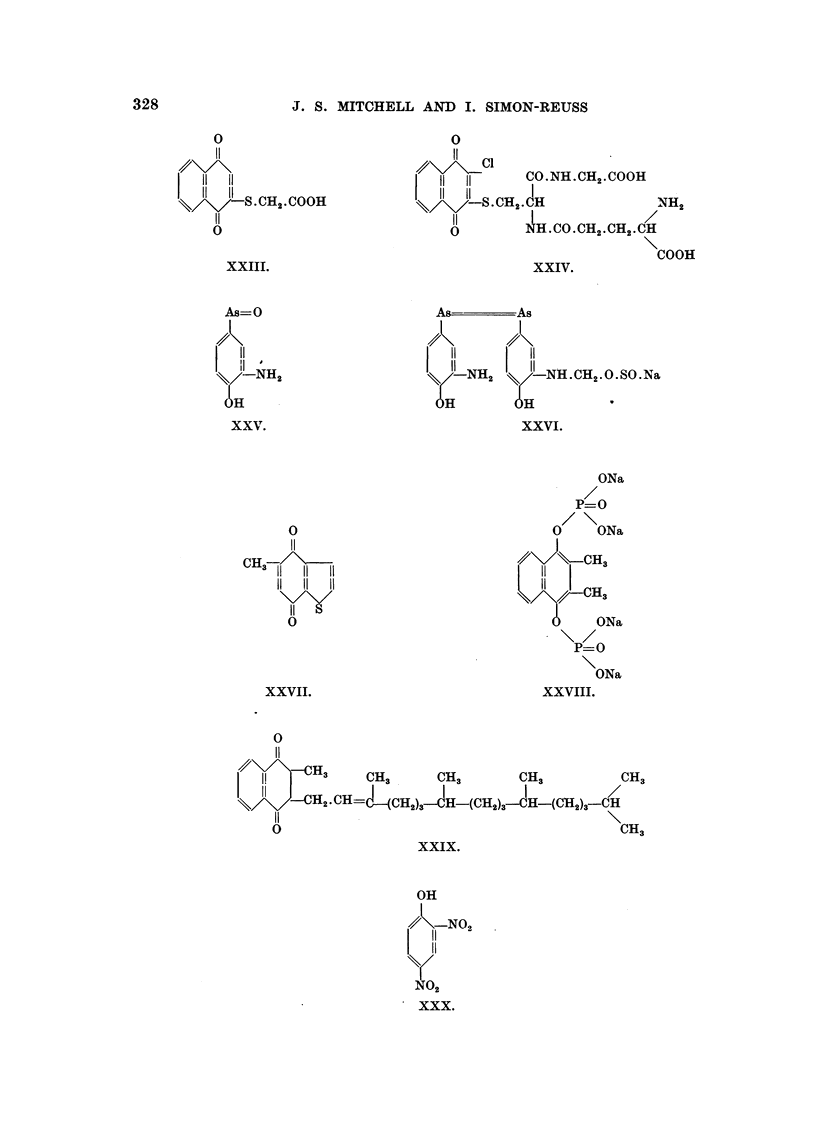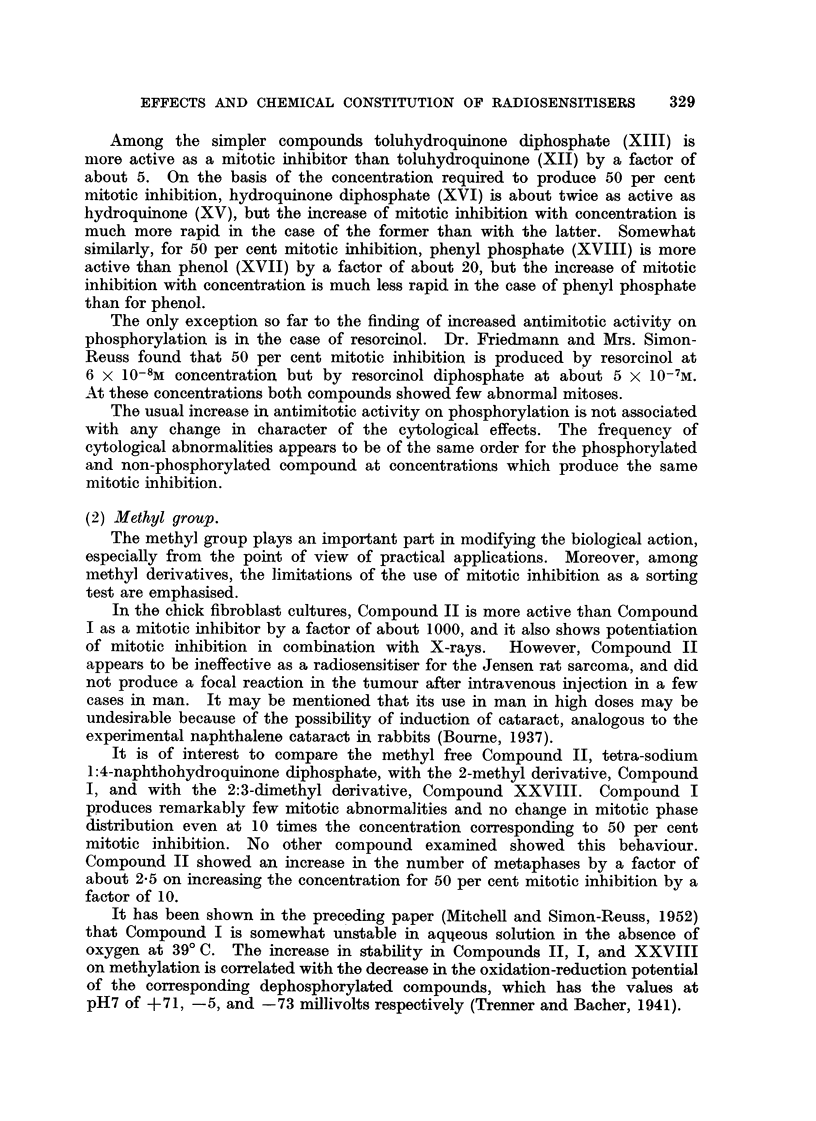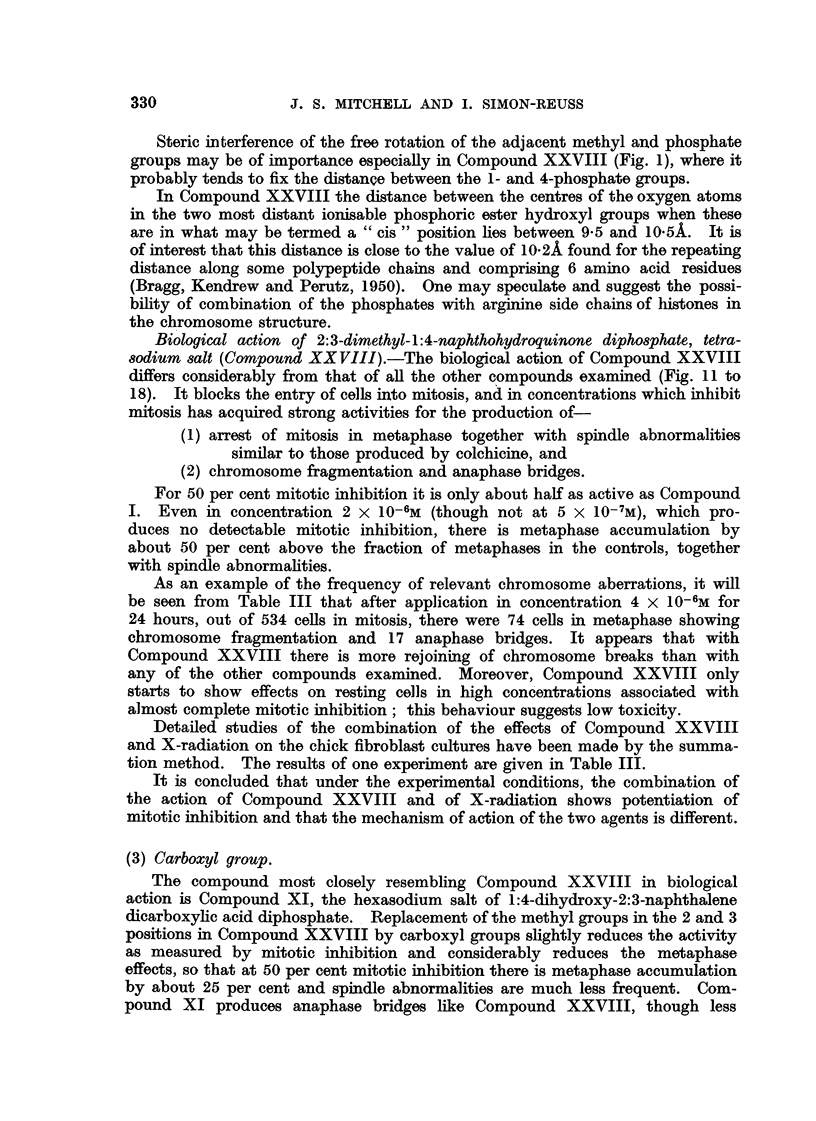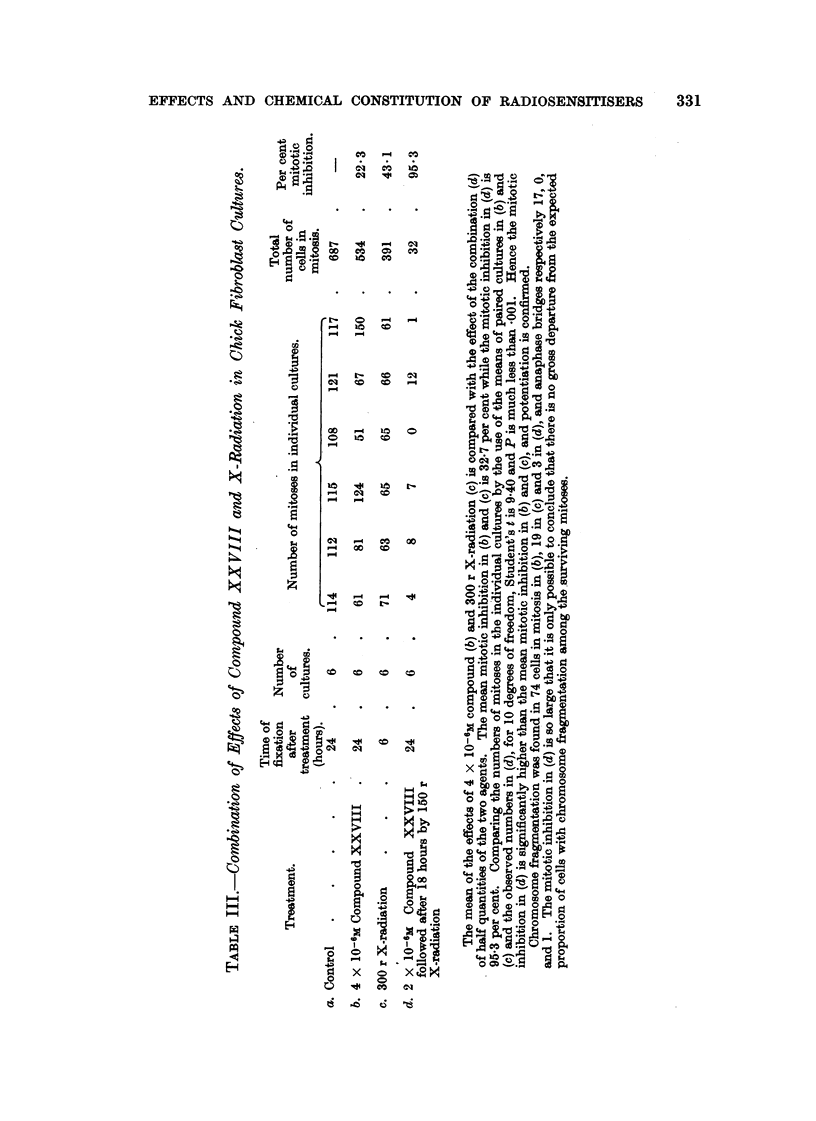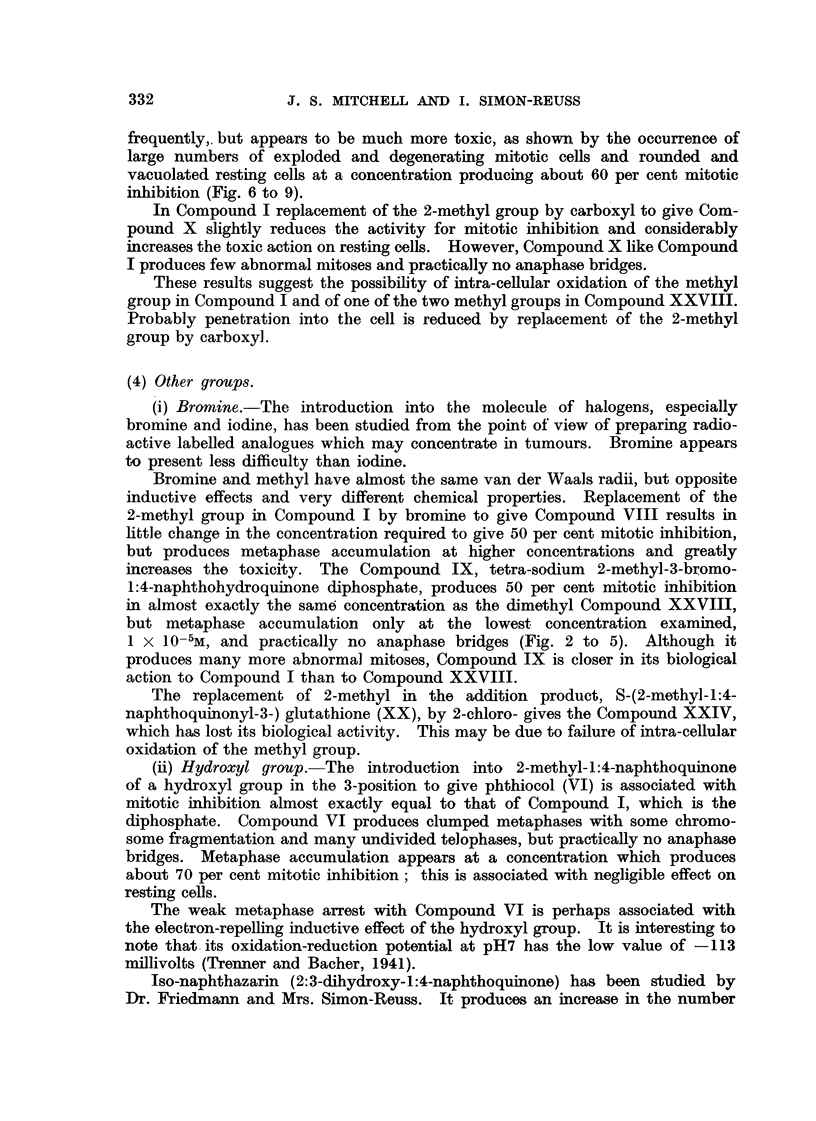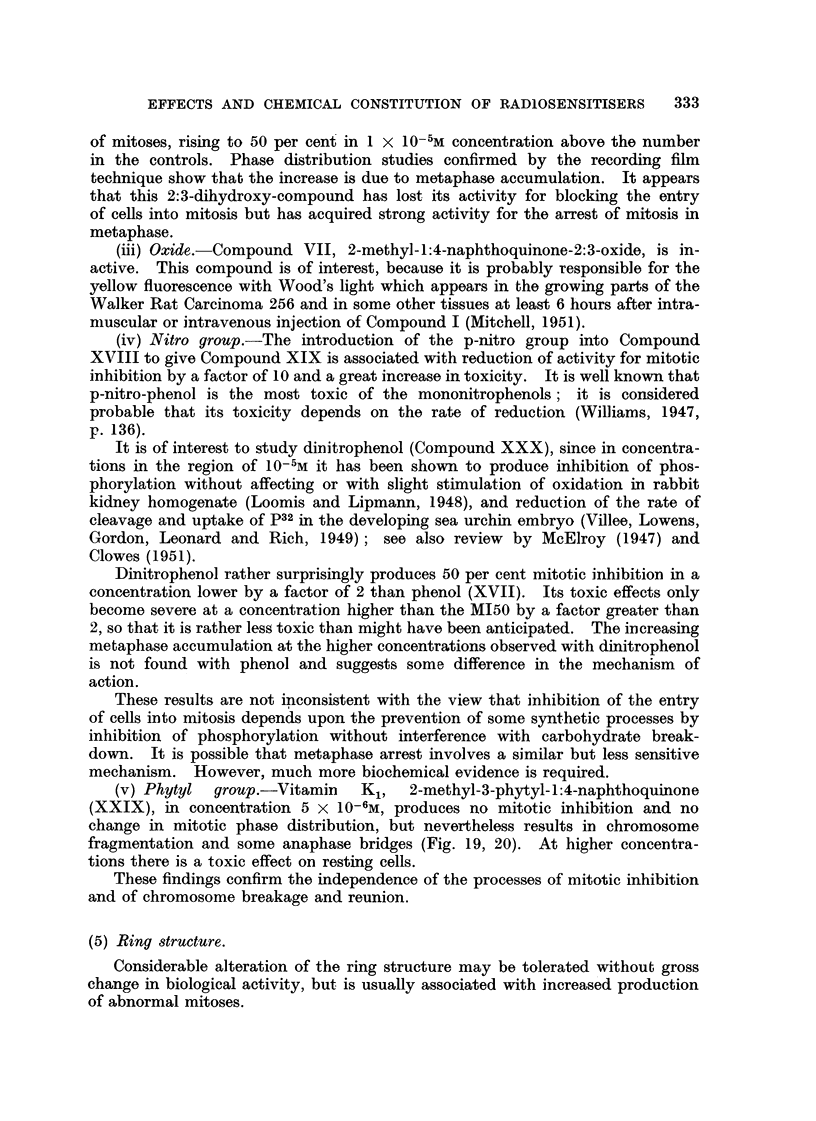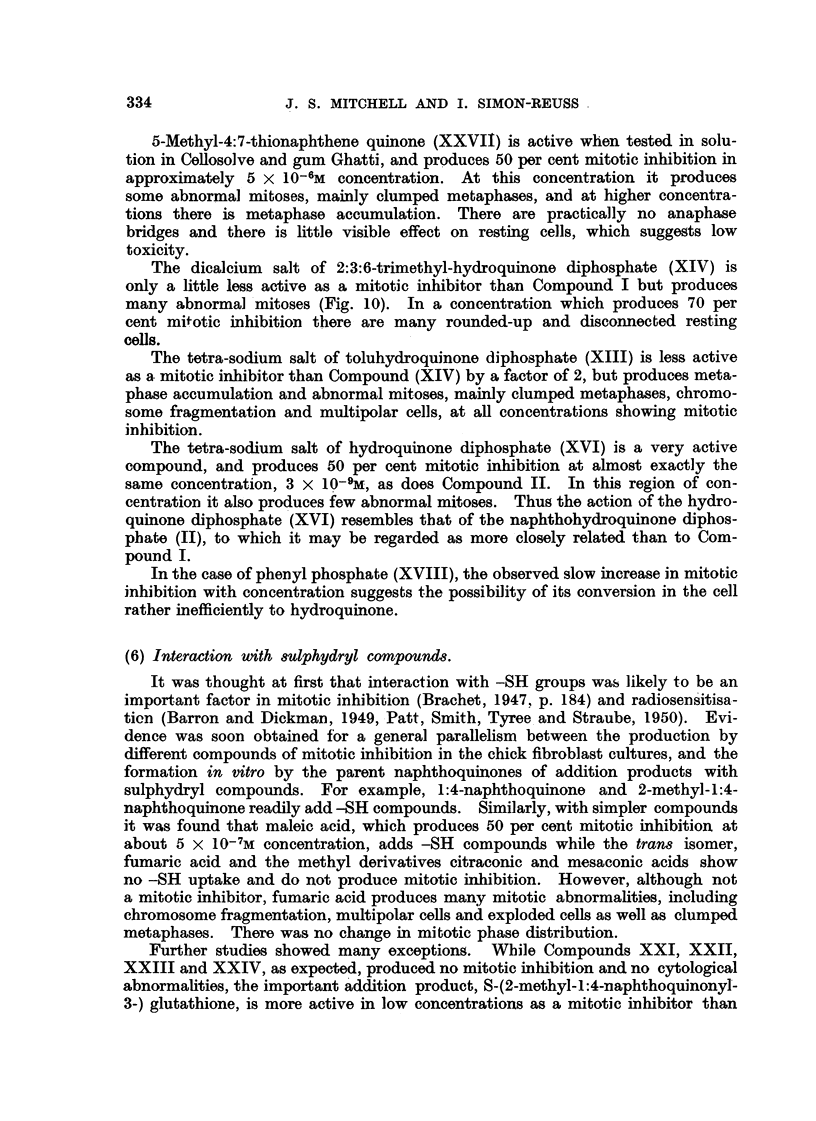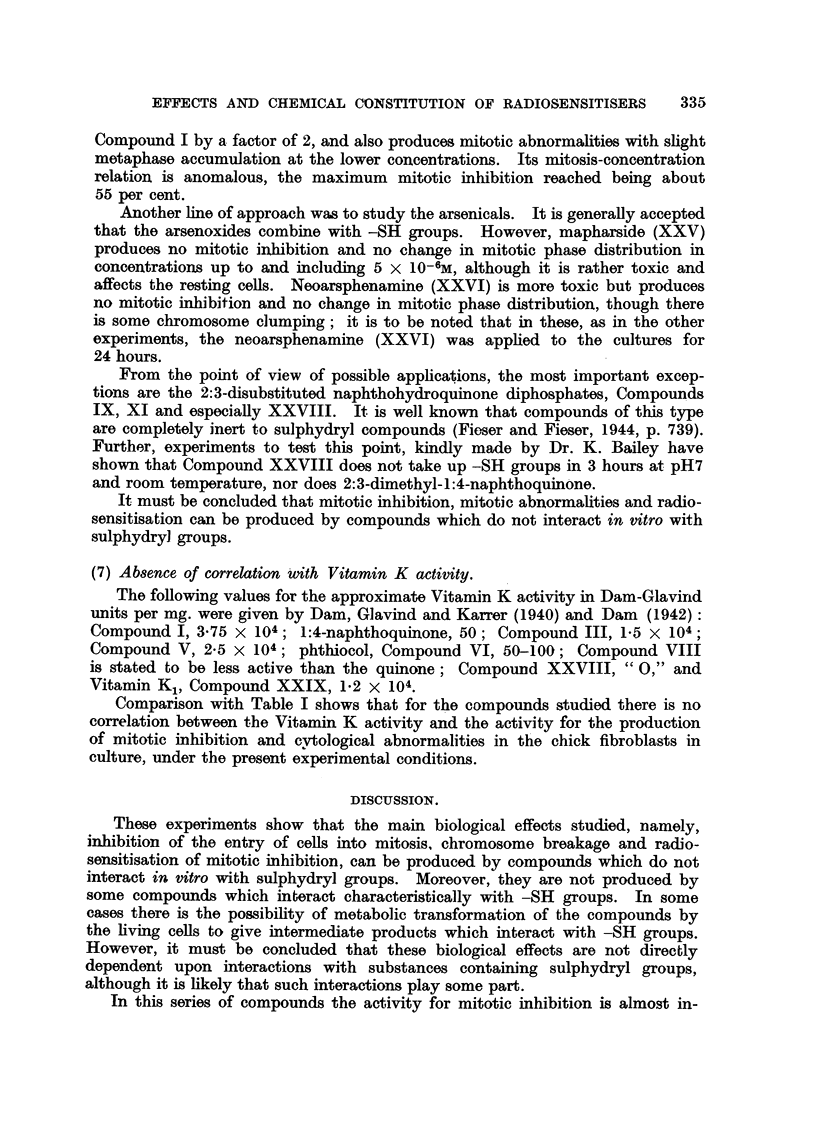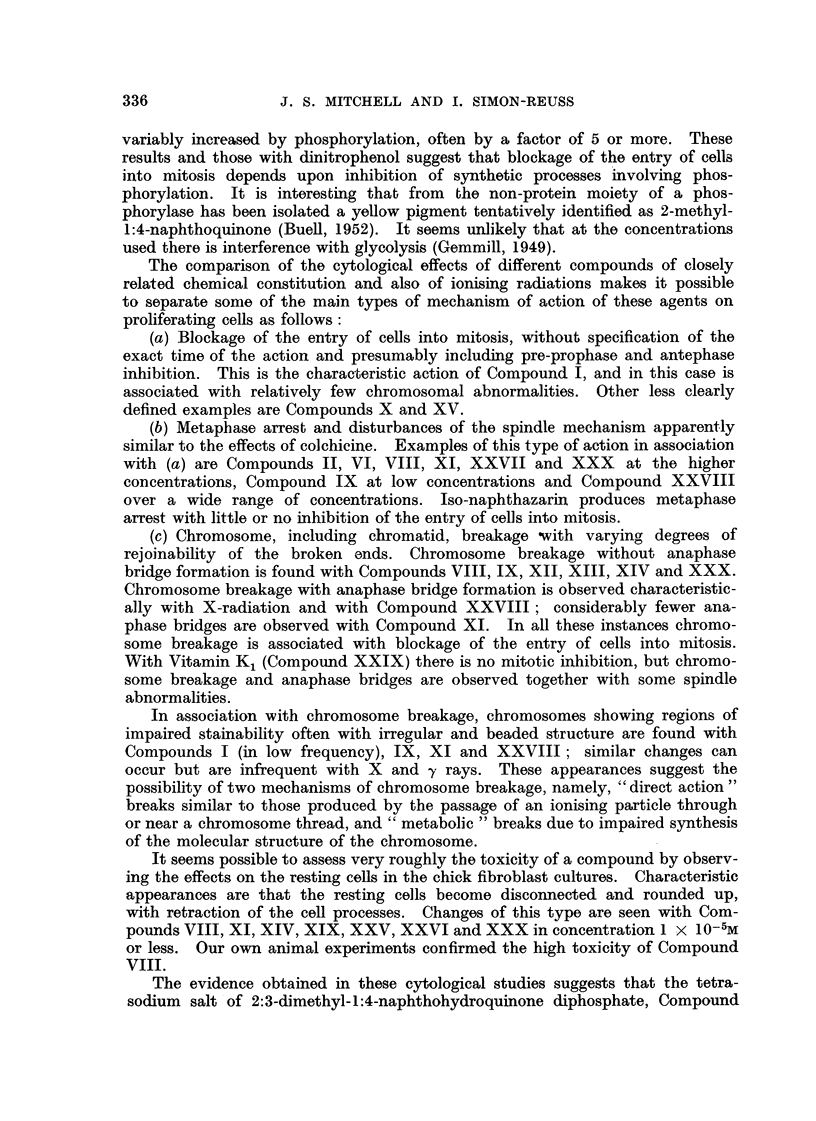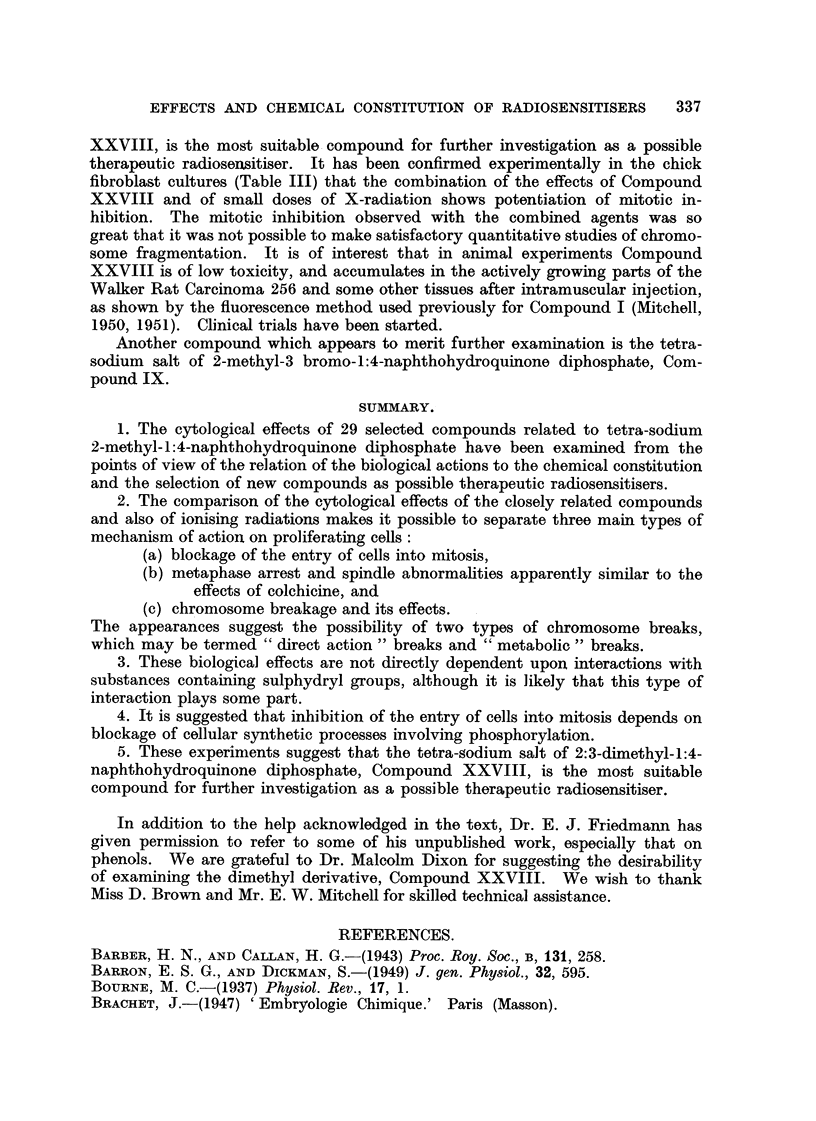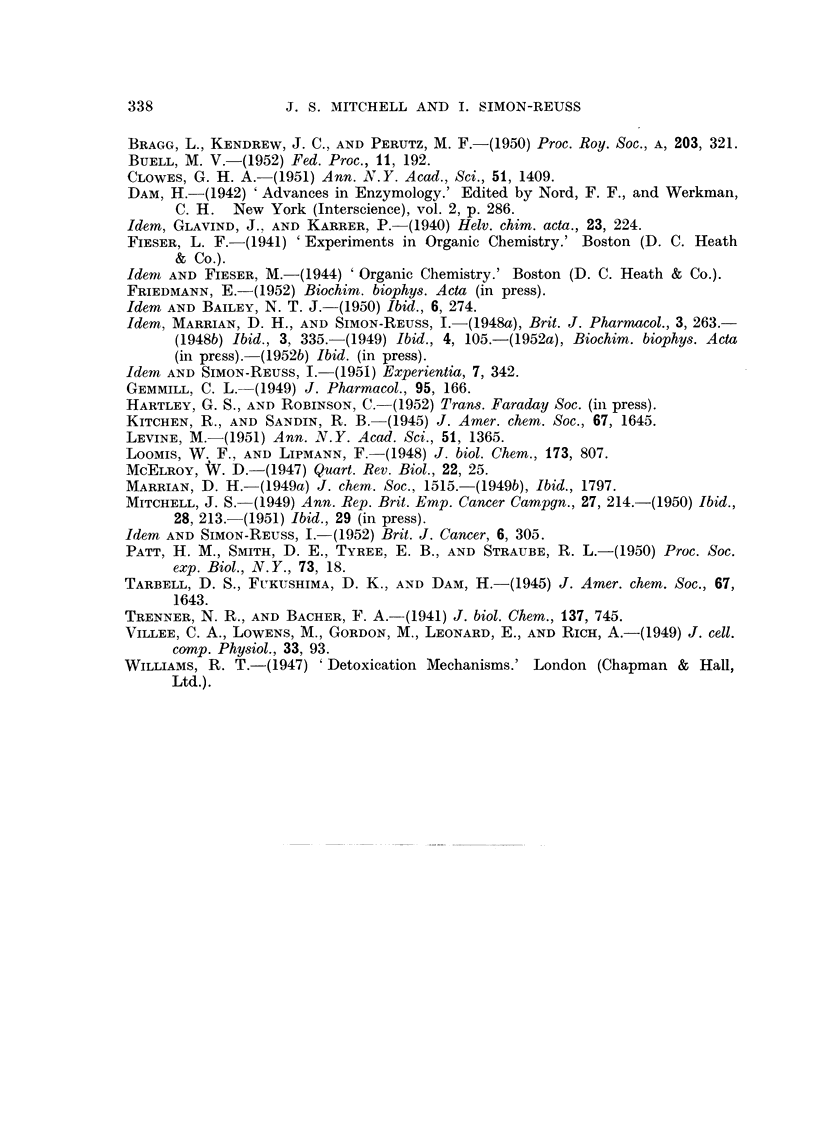# Experiments on the Mechanism of Action of Tetra-sodium 2-Methyl-1:4-Naphthohydroquinone Diphosphate as a Mitotic Inhibitor and Radiosensitiser, using the Technique of Tissue Culture: Relation between Cytological Effects and Chemical Constitution

**DOI:** 10.1038/bjc.1952.36

**Published:** 1952-09

**Authors:** J. S. Mitchell, I. Simon-Reuss

## Abstract

**Images:**


### EXPERIMENTS ON THE MECHANISM OF ACTION OF TETRA-SODIUM 2 - METHYL - 1 : 4 - NAPHTHOHYDROQUINONE DIPHOSPHATE AS A MITOTIC INHIBITOR AND RADIOSENSITISER, USING THE TECHNIQUE OF TISSUE CULTURE: RELATION BETWEEN CYTOLOGICAL EFFECTS AND CHEMICAL CONSTITUTION.

J. S. MITCHELL AND I. SIMON-REUSS.

*From the Department of Radiotherapeutics, University of Cambridge.*

Received for publication June 3, 1952.

THE study of the biological effects on chick fibroblast cultures has been used as the main sorting test for possible therapeutic radiosensitisers. It seems reasonable to select a compound which not only blocks the entry of cells into mitosis but also produces chromosome breakage, preferably together with anaphase bridges. Other desirable properties are low toxicity, stability and a molecular structure suggesting ease of penetration into cells. It seems probable that a colchicine-like action is not essential for radio-sensitisation. There appears to be no conclusive evidence that colchicine itself is of any practical value as a radiosensitiser (Levine, 1951).

Since 1946 an attempt has been made in collaboration with Dr. E. J. Friedmann and Dr. D. H. Marrian of this Department to study antimitotic action in relation to chemical structure. In all, about 100 compounds have been tested on chick fibroblast cultures. In addition to the reports by Mitchell (1949, 1950, 1951), a number of papers dealing mainly with mitotic inhibition have been published on the following groups of compounds and aspects of the problem: certain quinones (Friedmann, Marrian and Simon-Reuss, 1948*a*), sulphydryl addition compounds of some quinones and related compounds (Friedmann, Marrian and Simon-Reuss, 1948b), maleimide and related substances (Friedmann, Marrian and Simon-Reuss, 1949), the reactions of substituted maleimides with thiols (Marrian, 1949*a*), the condensation of N-substituted maleimides with thiourea (Marrian, 1949*b*), the action of 1:4-naphthohydroquinone diphosphate (Friedmann and Bailey, 1950), unsaturated imides with special reference to their reaction with sulphdryl groups (Friedmann, Marrian and Simon-Reuss, 1952*a*), halogen derivatives of the 1:4-naphthoquinone group and the maleic acid series (Friedmann, Marrian and Simon-Reuss, 1952*b*), and a spectrometric investigation of the interation of glutathione with maleimide and N-ethylmaleimide (Friedmann, 1952). As a different method of approach, studies are in progress on the antagonisation of the antimitotic action of tetra-sodium 2-methyl-1:4-naphthohydroquinone diphosphate (Compound I) by nucleotides, nucleosides, purines and pyrimidines and some other compounds (Mitchell, 1950, 1951).

This paper is an account of the cytological effects of a series of 29 selected compounds allied to tetra-sodium 2-methyl-1:4-naphthohydroquinone diphosphate (Compound I) from the points of view of—

(a) the relation of the chemical constitution to the biological action of Compound I, and

(b) the selection of new compounds as possible therapeutic radiosensitisers.

Although many of these compounds have been described previously from the point of view of mitotic inhibition and their preparation and chemical properties, little has been published about their cytological effects and new information is given about 12 compounds which we have examined.

### EXPERIMENTAL METHODS.

The experimental methods are those described by Mitchell and Simon-Reuss (1952). In the case of a few compounds of interest, experiments have been made to test for radiosensitisation in the tissue cultures, using the summation method.

Recently some water-insoluble compounds have been tested in solution in ethylene glycol mono-ethyl ether (Cellosolve) combined with gum Ghatti, using the method proposed by Friedmann and Simon-Reuss (1951). However, we have found that these agents in the concentrations used produce a significant increase in the mitotic inhibition observed at 6 hours after 300 r of X-radiation, so that caution is necessary in the application of this method to studies of radiosensitisation.

### *Preparations used.*

Most of the chemicals used in this investigation were either prepared or purified by Dr. E. J. Friedmann and Dr. D. H. Marrian. Details of preparations have been published as follows: Compounds II and IV (Friedmann, Marrian and Simon-Reuss, 1948*a*), Compounds XX–XXIII (Friedmann, Marrian and Simon-Reuss, 1948*b*), Compounds VIII, IX and XXIV (Friedmann, Marrian and Simon-Reuss 1952*a*).

We are indebted to Dr. F. Bergel, of Roche Products, Limited, for the following compounds and information about them: Compound X, $C_{11}H_5O_{10}P_2Na_5 . 4C_2H_5OH . 5H_2O$, mol. wt. 748·2, calc.: C, 30·5; H, 5·2; P, 8·3; found: C, 30·52; H, 3·1; P, 8·33, 8·20; Compound XI, $C_{12}H_4O_{12}P_2Na_6 . 7H_2O$, mol. wt. 666·2, calc.: C, 21·7; H, 2·7; P, 9·3; found: C, 21·9; H, 3·3; P, 9·2, 9·4; Compound XIV, $C_9H_{10}O_8P_2Ca_2 . H_2O . \frac{1}{2} C_2H_5OH$, mol. wt. 429·41, calc.: C, 28·0; H, 3·5; P, 14·45, found: C, 28·67; H, 3·27; P, 14·37, 14·10. The specimen of vitamin $K_1$ was kindly given by Dr. F. Wrigley of Roche Products, Limited.

Compound XXVII, described by Tarbell, Fukushima and Dam (1945) and Kitchen and Sandin (1945) was prepared by Dr. Friedmann.

Compound XXVIII was prepared by Dr. Marrian, who has kindly given us the following information about the di-barium salt of 2:3-dimethyl-1:4-naphthohydroquinone diphosphate: Colourless prisms, $C_{12}H_{10}O_8P_2Ba_2 . 5H_2O$, calc.: C, 20·4; H, 2·82; P, 8·75; loss of $5H_2O$, 12·7 per cent; found: C, 20·0; H, 2·72; P. 8·55; loss at 120° 11·6 per cent; at pH2 the molecular extinction coefficient has the values $5·42 \times 10^3$ at 290 m$\mu$. and $6·24 \times 10^4$ at 230 m$\mu$.

### RESULTS.

The effects of the selected chemical agents on the chick fibroblasts in culture are summarised in Table I. The cytological effects are considered in relation to those of Compound I. Of especial interest from the point of view of the selection of compounds as possible therapeutic radiosensitisers is the appearance of mitotic abnormalities in concentrations which produce mitotic inhibition without gross toxic effects on the resting cells. The most important abnormalities studied are chromosome fragmentation, anaphase bridges, metaphase chromosomes with regions of impaired stainability and with irregular and beaded structure, metaphases with clumping of the chromosomes, arrested metaphases and other spindle abnormalities, of which many of the types described by Barber and Callan (1943) have been noted. As already discussed (Mitchell and Simon-Reuss, 1952), it seems likely that the most relevant cytological abnormalities are chromosome fragmentation, anaphase bridges and chromosome aberrations in general. Examples of the various types of cytological effects observed with this series of compounds are shown in Fig. 2 to 20. It is evident that none of the abnormalities are specific for particular compounds, but in the figures an attempt is made to select the commonest or most characteristic cytological changes associated with the more interesting compounds.

The formulae of the compounds are given in Table II. These " pictorial " representations of the chemical structure have been supplemented by studies of more accurate molecular models of the " space filling " type (Hartley and Robinson, 1952), e.g., Fig. 1 shows photographs of models of the molecule of Compound XXVIII.

### *Discussion of Cytological Action in Relation to Chemical Constitution.*

The main factors to be considered are :

(1) the importance of phosphorylation,
(2) the influence of the methyl group,
(3) the role of the carboxyl group,
(4) the influence of other groups especially bromine, hydroxyl and oxide,
(5) alteration of the ring structure,
(6) interactions with sulphydryl groups, and
(7) comparison with vitamin K activity.

(1) *Phosphate groups.*

Phosphorylation almost invariably increases the antimitotic activity.

2-Methyl-1:4-naphthoquinone (V) produces no detectable mitotic inhibition in concentration $1 \times 10^{-6}$M, about 7 per cent mitotic inhibition in $5 \times 10^{-6}$M and 32 per cent mitotic inhibition in $1 \times 10^{-5}$M when tested in solution in Cellosolve and gum Ghatti, the presence of which does not affect the Compound I. There are toxic effects on the resting cells at $1 \times 10^{-5}$M concentration and these increase rapidly with increasing concentration, so that it is impossible to determine directly the concentration corresponding to 50 per cent mitotic inhibition (MI50) for this compound. A specimen of 2-methyl-1:4-naphthohydroquinone, which is a poorly characterised compound with no sharp melting-point (Fieser, 1941, p. 235), also produced no significant mitotic inhibition in concentration $10^{-6}$–$10^{-5}$M when tested in Cellosolve and gum Ghatti. Thus phosphorylation increases the antimitotic activity under the experimental conditions by a factor of at least 5. Moreover, replacement of the two phosphate groups in Compound I by two succinate groups leads to Compound III which is unstable but almost certainly of very low activity. Similarly in relation to Compound II, 1:4-naphthohydroquinone monohydrogen succinate (IV) is of low activity. It is possible, though unlikely, that the cultures and the medium lack enzymes capable of hydrolysing the succinates.

---

### EXPLANATION OF PLATES.

FIG. 1.—Photograph of model of molecule of tetra-sodium 2:3-dimethyl-1:4-naphthohydroquinone diphosphate (Compound XXVIII).

The atomic models are of the "space filling" type (Hartley and Robinson, 1952). The naphthalene ring is viewed laterally. Nearest the scale are the two methyl groups in the 2- and 3- positions. The phosphoric ester groups are seen laterally in the 1- and 4- positions. The P atoms are largely covered by the O atoms (white). The centres of the most lateral O atoms, which are covered by the small grey H atoms, are separated by a distance which lies between 9·5 and 10·5 Å.

FIG. 2 to 5.—Effect of tetra-sodium salt of 2-methyl-3-bromo-1:4-naphthohydroquinone diphosphate (Compound IX) on chick fibroblast cultures. Concentration $5 \times 10^{-6}$M. Fixed after 24 hours. × 1200.

FIG. 2.—Arrested metaphases. Upper cell shows a chromosome with regions of impaired stainability.

FIG. 3.—Metaphase with gross clumping of chromosomes.

FIG. 4.—Anaphase with irregular distribution of chromosomes.

FIG. 5.—Anaphase with incompact spindle and chromosome bridges.

FIG. 6 to 9.—Effects of hexa-sodium salt of 1:4-dihydroxy-2:3-naphthalene dicarboxylic acid diphosphate (Compound XI) on chick fibroblast cultures. Concentration $6 \times 10^{-6}$M. Fixed after 24 hours.

FIG. 6.—Enlarged cell. Metaphase with irregular distribution of chromosomes, partial chromosome clumping and some chromosomes showing regions of defective stainability. × 1200.

FIG. 7.—Metaphase with gross clumping of chromosomes. × 1200.

FIG. 8.—Degenerate cell and débris of many breaking-down cells. × 1200.

FIG. 9.—Enlarged cell, probably anaphase with irregular tripolar spindle, anaphase bridge and chromosome fragmentation. × 875.

FIG. 10.—Effect of dicalcium salt of 2:3:6-trimethyl-hydroquinone diphosphate (Compound XIV) on chick fibroblast cultures. Concentration $6 \times 10^{-6}$M. Fixed after 24 hours. Enlarged cell. Tripolar metaphase. × 1200.

FIG. 11–18.—Effects of tetra-sodium 2:3-dimethyl-1:4-naphthohydroquinone diphosphate (Compound XXVIII) on chick fibroblast cultures. Fixed after 24 hours.

FIG. 11.—Control. × 185.

FIG. 12.—At 24 hours after application of compound XXVIII in concentration $6 \times 10^{-6}$M. Metaphase accumulation. × 185.

FIG. 13–18.—At 24 hours after application in concentration $4 \times 10^{-6}$M. × 1175.

FIG. 13.—Anaphase bridges, chromosome fragments and cytoplasmic vacuolation outside the spindle area.

FIG. 14.—Anaphase bridges.

FIG. 15.—Two cells showing metaphase arrest with a clearly defined spindle, some irregularity of chromosome arrangement and cytoplasmic vacuolation outside the spindle area.

FIG. 16.—Three cells in metaphase showing gross clumping of chromosomes.

FIG. 17.—Distorted metaphase with incomplete spindle formation and cytoplasmic vacuolation.

FIG. 18.—Enlarged cell; prometaphase with irregular arrangement of chromosomes, and chromosomes showing regions of defective staining.

FIG. 19, 20.—Effect of Vitamin $K_1$ (Compound XXIX) on chick fibroblast cultures. Concentration $5 \times 10^{-6}$M. in Cellosolve and gum Ghatti. Fixed after 24 hours. × 1175.

FIG. 19.—Metaphase with partial chromosome clumping.

FIG. 20.—Anaphase with spindle abnormality and chromosome bridge and fragments.

1

2

3

4

Mitchell and Simon-Reuss.

Mitchell and Simon-Reuss.

13

14

15

16

17

18

19

20

### Table II.

I.

II.

III.

IV.

V.

VI.

VII.

VIII.

IX.

X.

XI.

XII.

XIII.

XIV.

XV.

XVI.

XVII.

XVIII.

XIX.

XX.

XXI.

XXII.

XXIII.

XXIV.

XXV.

XXVI.

XXVII.

XXVIII.

XXIX.

XXX.

Among the simpler compounds toluhydroquinone diphosphate (XIII) is more active as a mitotic inhibitor than toluhydroquinone (XII) by a factor of about 5. On the basis of the concentration required to produce 50 per cent mitotic inhibition, hydroquinone diphosphate (XVI) is about twice as active as hydroquinone (XV), but the increase of mitotic inhibition with concentration is much more rapid in the case of the former than with the latter. Somewhat similarly, for 50 per cent mitotic inhibition, phenyl phosphate (XVIII) is more active than phenol (XVII) by a factor of about 20, but the increase of mitotic inhibition with concentration is much less rapid in the case of phenyl phosphate than for phenol.

The only exception so far to the finding of increased antimitotic activity on phosphorylation is in the case of resorcinol. Dr. Friedmann and Mrs. Simon-Reuss found that 50 per cent mitotic inhibition is produced by resorcinol at $6 \times 10^{-8}$M concentration but by resorcinol diphosphate at about $5 \times 10^{-7}$M. At these concentrations both compounds showed few abnormal mitoses.

The usual increase in antimitotic activity on phosphorylation is not associated with any change in character of the cytological effects. The frequency of cytological abnormalities appears to be of the same order for the phosphorylated and non-phosphorylated compound at concentrations which produce the same mitotic inhibition.

(2) *Methyl group.*

The methyl group plays an important part in modifying the biological action, especially from the point of view of practical applications. Moreover, among methyl derivatives, the limitations of the use of mitotic inhibition as a sorting test are emphasised.

In the chick fibroblast cultures, Compound II is more active than Compound I as a mitotic inhibitor by a factor of about 1000, and it also shows potentiation of mitotic inhibition in combination with X-rays. However, Compound II appears to be ineffective as a radiosensitiser for the Jensen rat sarcoma, and did not produce a focal reaction in the tumour after intravenous injection in a few cases in man. It may be mentioned that its use in man in high doses may be undesirable because of the possibility of induction of cataract, analogous to the experimental naphthalene cataract in rabbits (Bourne, 1937).

It is of interest to compare the methyl free Compound II, tetra-sodium 1:4-naphthohydroquinone diphosphate, with the 2-methyl derivative, Compound I, and with the 2:3-dimethyl derivative, Compound XXVIII. Compound I produces remarkably few mitotic abnormalities and no change in mitotic phase distribution even at 10 times the concentration corresponding to 50 per cent mitotic inhibition. No other compound examined showed this behaviour. Compound II showed an increase in the number of metaphases by a factor of about 2·5 on increasing the concentration for 50 per cent mitotic inhibition by a factor of 10.

It has been shown in the preceding paper (Mitchell and Simon-Reuss, 1952) that Compound I is somewhat unstable in aqueous solution in the absence of oxygen at 39° C. The increase in stability in Compounds II, I, and XXVIII on methylation is correlated with the decrease in the oxidation-reduction potential of the corresponding dephosphorylated compounds, which has the values at pH7 of +71, —5, and —73 millivolts respectively (Trenner and Bacher, 1941).

Steric interference of the free rotation of the adjacent methyl and phosphate groups may be of importance especially in Compound XXVIII (Fig. 1), where it probably tends to fix the distance between the 1- and 4-phosphate groups.

In Compound XXVIII the distance between the centres of the oxygen atoms in the two most distant ionisable phosphoric ester hydroxyl groups when these are in what may be termed a " cis " position lies between 9·5 and 10·5Å. It is of interest that this distance is close to the value of 10·2Å found for the repeating distance along some polypeptide chains and comprising 6 amino acid residues (Bragg, Kendrew and Perutz, 1950). One may speculate and suggest the possibility of combination of the phosphates with arginine side chains of histones in the chromosome structure.

*Biological action of* 2:3-*dimethyl*-1:4-*naphthohydroquinone diphosphate, tetrasodium salt* (*Compound XXVIII*).—The biological action of Compound XXVIII differs considerably from that of all the other compounds examined (Fig. 11 to 18). It blocks the entry of cells into mitosis, and in concentrations which inhibit mitosis has acquired strong activities for the production of—

(1) arrest of mitosis in metaphase together with spindle abnormalities similar to those produced by colchicine, and
(2) chromosome fragmentation and anaphase bridges.

For 50 per cent mitotic inhibition it is only about half as active as Compound I. Even in concentration $2 \times 10^{-6}$M (though not at $5 \times 10^{-7}$M), which produces no detectable mitotic inhibition, there is metaphase accumulation by about 50 per cent above the fraction of metaphases in the controls, together with spindle abnormalities.

As an example of the frequency of relevant chromosome aberrations, it will be seen from Table III that after application in concentration $4 \times 10^{-6}$M for 24 hours, out of 534 cells in mitosis, there were 74 cells in metaphase showing chromosome fragmentation and 17 anaphase bridges. It appears that with Compound XXVIII there is more rejoining of chromosome breaks than with any of the other compounds examined. Moreover, Compound XXVIII only starts to show effects on resting cells in high concentrations associated with almost complete mitotic inhibition ; this behaviour suggests low toxicity.

Detailed studies of the combination of the effects of Compound XXVIII and X-radiation on the chick fibroblast cultures have been made by the summation method. The results of one experiment are given in Table III.

It is concluded that under the experimental conditions, the combination of the action of Compound XXVIII and of X-radiation shows potentiation of mitotic inhibition and that the mechanism of action of the two agents is different.

(3) *Carboxyl group.*

The compound most closely resembling Compound XXVIII in biological action is Compound XI, the hexasodium salt of 1:4-dihydroxy-2:3-naphthalene dicarboxylic acid diphosphate. Replacement of the methyl groups in the 2 and 3 positions in Compound XXVIII by carboxyl groups slightly reduces the activity as measured by mitotic inhibition and considerably reduces the metaphase effects, so that at 50 per cent mitotic inhibition there is metaphase accumulation by about 25 per cent and spindle abnormalities are much less frequent. Compound XI produces anaphase bridges like Compound XXVIII, though less frequently, but appears to be much more toxic, as shown by the occurrence of large numbers of exploded and degenerating mitotic cells and rounded and vacuolated resting cells at a concentration producing about 60 per cent mitotic inhibition (Fig. 6 to 9).

In Compound I replacement of the 2-methyl group by carboxyl to give Compound X slightly reduces the activity for mitotic inhibition and considerably increases the toxic action on resting cells. However, Compound X like Compound I produces few abnormal mitoses and practically no anaphase bridges.

These results suggest the possibility of intra-cellular oxidation of the methyl group in Compound I and of one of the two methyl groups in Compound XXVIII. Probably penetration into the cell is reduced by replacement of the 2-methyl group by carboxyl.

(4) *Other groups.*

(i) *Bromine.*—The introduction into the molecule of halogens, especially bromine and iodine, has been studied from the point of view of preparing radioactive labelled analogues which may concentrate in tumours. Bromine appears to present less difficulty than iodine.

Bromine and methyl have almost the same van der Waals radii, but opposite inductive effects and very different chemical properties. Replacement of the 2-methyl group in Compound I by bromine to give Compound VIII results in little change in the concentration required to give 50 per cent mitotic inhibition, but produces metaphase accumulation at higher concentrations and greatly increases the toxicity. The Compound IX, tetra-sodium 2-methyl-3-bromo-1:4-naphthohydroquinone diphosphate, produces 50 per cent mitotic inhibition in almost exactly the same concentration as the dimethyl Compound XXVIII, but metaphase accumulation only at the lowest concentration examined, $1 \times 10^{-5}$M, and practically no anaphase bridges (Fig. 2 to 5). Although it produces many more abnormal mitoses, Compound IX is closer in its biological action to Compound I than to Compound XXVIII.

The replacement of 2-methyl in the addition product, S-(2-methyl-1:4-naphthoquinonyl-3-) glutathione (XX), by 2-chloro- gives the Compound XXIV, which has lost its biological activity. This may be due to failure of intra-cellular oxidation of the methyl group.

(ii) *Hydroxyl group.*—The introduction into 2-methyl-1:4-naphthoquinone of a hydroxyl group in the 3-position to give phthiocol (VI) is associated with mitotic inhibition almost exactly equal to that of Compound I, which is the diphosphate. Compound VI produces clumped metaphases with some chromosome fragmentation and many undivided telophases, but practically no anaphase bridges. Metaphase accumulation appears at a concentration which produces about 70 per cent mitotic inhibition ; this is associated with negligible effect on resting cells.

The weak metaphase arrest with Compound VI is perhaps associated with the electron-repelling inductive effect of the hydroxyl group. It is interesting to note that its oxidation-reduction potential at pH7 has the low value of —113 millivolts (Trenner and Bacher, 1941).

Iso-naphthazarin (2:3-dihydroxy-1:4-naphthoquinone) has been studied by Dr. Friedmann and Mrs. Simon-Reuss. It produces an increase in the number of mitoses, rising to 50 per cent in $1 \times 10^{-5}$M concentration above the number in the controls. Phase distribution studies confirmed by the recording film technique show that the increase is due to metaphase accumulation. It appears that this 2:3-dihydroxy-compound has lost its activity for blocking the entry of cells into mitosis but has acquired strong activity for the arrest of mitosis in metaphase.

(iii) *Oxide.*—Compound VII, 2-methyl-1:4-naphthoquinone-2:3-oxide, is inactive. This compound is of interest, because it is probably responsible for the yellow fluorescence with Wood's light which appears in the growing parts of the Walker Rat Carcinoma 256 and in some other tissues at least 6 hours after intramuscular or intravenous injection of Compound I (Mitchell, 1951).

(iv) *Nitro group.*—The introduction of the p-nitro group into Compound XVIII to give Compound XIX is associated with reduction of activity for mitotic inhibition by a factor of 10 and a great increase in toxicity. It is well known that p-nitro-phenol is the most toxic of the mononitrophenols; it is considered probable that its toxicity depends on the rate of reduction (Williams, 1947, p. 136).

It is of interest to study dinitrophenol (Compound XXX), since in concentrations in the region of $10^{-5}$M it has been shown to produce inhibition of phosphorylation without affecting or with slight stimulation of oxidation in rabbit kidney homogenate (Loomis and Lipmann, 1948), and reduction of the rate of cleavage and uptake of $P^{32}$ in the developing sea urchin embryo (Villee, Lowens, Gordon, Leonard and Rich, 1949); see also review by McElroy (1947) and Clowes (1951).

Dinitrophenol rather surprisingly produces 50 per cent mitotic inhibition in a concentration lower by a factor of 2 than phenol (XVII). Its toxic effects only become severe at a concentration higher than the MI50 by a factor greater than 2, so that it is rather less toxic than might have been anticipated. The increasing metaphase accumulation at the higher concentrations observed with dinitrophenol is not found with phenol and suggests some difference in the mechanism of action.

These results are not inconsistent with the view that inhibition of the entry of cells into mitosis depends upon the prevention of some synthetic processes by inhibition of phosphorylation without interference with carbohydrate breakdown. It is possible that metaphase arrest involves a similar but less sensitive mechanism. However, much more biochemical evidence is required.

(v) *Phytyl group.*—Vitamin $K_1$, 2-methyl-3-phytyl-1:4-naphthoquinone (XXIX), in concentration $5 \times 10^{-6}$M, produces no mitotic inhibition and no change in mitotic phase distribution, but nevertheless results in chromosome fragmentation and some anaphase bridges (Fig. 19, 20). At higher concentrations there is a toxic effect on resting cells.

These findings confirm the independence of the processes of mitotic inhibition and of chromosome breakage and reunion.

(5) *Ring structure.*

Considerable alteration of the ring structure may be tolerated without gross change in biological activity, but is usually associated with increased production of abnormal mitoses.

5-Methyl-4:7-thionaphthene quinone (XXVII) is active when tested in solution in Cellosolve and gum Ghatti, and produces 50 per cent mitotic inhibition in approximately $5 \times 10^{-6}$M concentration. At this concentration it produces some abnormal mitoses, mainly clumped metaphases, and at higher concentrations there is metaphase accumulation. There are practically no anaphase bridges and there is little visible effect on resting cells, which suggests low toxicity.

The dicalcium salt of 2:3:6-trimethyl-hydroquinone diphosphate (XIV) is only a little less active as a mitotic inhibitor than Compound I but produces many abnormal mitoses (Fig. 10). In a concentration which produces 70 per cent mitotic inhibition there are many rounded-up and disconnected resting cells.

The tetra-sodium salt of toluhydroquinone diphosphate (XIII) is less active as a mitotic inhibitor than Compound (XIV) by a factor of 2, but produces metaphase accumulation and abnormal mitoses, mainly clumped metaphases, chromosome fragmentation and multipolar cells, at all concentrations showing mitotic inhibition.

The tetra-sodium salt of hydroquinone diphosphate (XVI) is a very active compound, and produces 50 per cent mitotic inhibition at almost exactly the same concentration, $3 \times 10^{-9}$M, as does Compound II. In this region of concentration it also produces few abnormal mitoses. Thus the action of the hydroquinone diphosphate (XVI) resembles that of the naphthohydroquinone diphosphate (II), to which it may be regarded as more closely related than to Compound I.

In the case of phenyl phosphate (XVIII), the observed slow increase in mitotic inhibition with concentration suggests the possibility of its conversion in the cell rather inefficiently to hydroquinone.

### (6) *Interaction with sulphydryl compounds.*

It was thought at first that interaction with –SH groups was likely to be an important factor in mitotic inhibition (Brachet, 1947, p. 184) and radiosensitisation (Barron and Dickman, 1949, Patt, Smith, Tyree and Straube, 1950). Evidence was soon obtained for a general parallelism between the production by different compounds of mitotic inhibition in the chick fibroblast cultures, and the formation *in vitro* by the parent naphthoquinones of addition products with sulphydryl compounds. For example, 1:4-naphthoquinone and 2-methyl-1:4-naphthoquinone readily add –SH compounds. Similarly, with simpler compounds it was found that maleic acid, which produces 50 per cent mitotic inhibition at about $5 \times 10^{-7}$M concentration, adds –SH compounds while the *trans* isomer, fumaric acid and the methyl derivatives citraconic and mesaconic acids show no –SH uptake and do not produce mitotic inhibition. However, although not a mitotic inhibitor, fumaric acid produces many mitotic abnormalities, including chromosome fragmentation, multipolar cells and exploded cells as well as clumped metaphases. There was no change in mitotic phase distribution.

Further studies showed many exceptions. While Compounds XXI, XXII, XXIII and XXIV, as expected, produced no mitotic inhibition and no cytological abnormalities, the important addition product, S-(2-methyl-1:4-naphthoquinonyl-3-) glutathione, is more active in low concentrations as a mitotic inhibitor than

Compound I by a factor of 2, and also produces mitotic abnormalities with slight metaphase accumulation at the lower concentrations. Its mitosis-concentration relation is anomalous, the maximum mitotic inhibition reached being about 55 per cent.

Another line of approach was to study the arsenicals. It is generally accepted that the arsenoxides combine with –SH groups. However, mapharside (XXV) produces no mitotic inhibition and no change in mitotic phase distribution in concentrations up to and including $5 \times 10^{-6}$M, although it is rather toxic and affects the resting cells. Neoarsphenamine (XXVI) is more toxic but produces no mitotic inhibition and no change in mitotic phase distribution, though there is some chromosome clumping ; it is to be noted that in these, as in the other experiments, the neoarsphenamine (XXVI) was applied to the cultures for 24 hours.

From the point of view of possible applications, the most important exceptions are the 2:3-disubstituted naphthohydroquinone diphosphates, Compounds IX, XI and especially XXVIII. It is well known that compounds of this type are completely inert to sulphydryl compounds (Fieser and Fieser, 1944, p. 739). Further, experiments to test this point, kindly made by Dr. K. Bailey have shown that Compound XXVIII does not take up –SH groups in 3 hours at pH7 and room temperature, nor does 2:3-dimethyl-1:4-naphthoquinone.

It must be concluded that mitotic inhibition, mitotic abnormalities and radiosensitisation can be produced by compounds which do not interact *in vitro* with sulphydryl groups.

(7) *Absence of correlation with Vitamin K activity.*

The following values for the approximate Vitamin K activity in Dam-Glavind units per mg. were given by Dam, Glavind and Karrer (1940) and Dam (1942) : Compound I, $3{\cdot}75 \times 10^4$ ; 1:4-naphthoquinone, 50 ; Compound III, $1{\cdot}5 \times 10^4$ ; Compound V, $2{\cdot}5 \times 10^4$ ; phthiocol, Compound VI, 50–100 ; Compound VIII is stated to be less active than the quinone ; Compound XXVIII, " O," and Vitamin $K_1$, Compound XXIX, $1{\cdot}2 \times 10^4$.

Comparison with Table I shows that for the compounds studied there is no correlation between the Vitamin K activity and the activity for the production of mitotic inhibition and cytological abnormalities in the chick fibroblasts in culture, under the present experimental conditions.

### DISCUSSION.

These experiments show that the main biological effects studied, namely, inhibition of the entry of cells into mitosis, chromosome breakage and radiosensitisation of mitotic inhibition, can be produced by compounds which do not interact *in vitro* with sulphydryl groups. Moreover, they are not produced by some compounds which interact characteristically with –SH groups. In some cases there is the possibility of metabolic transformation of the compounds by the living cells to give intermediate products which interact with –SH groups. However, it must be concluded that these biological effects are not directly dependent upon interactions with substances containing sulphydryl groups, although it is likely that such interactions play some part.

In this series of compounds the activity for mitotic inhibition is almost invariably increased by phosphorylation, often by a factor of 5 or more. These results and those with dinitrophenol suggest that blockage of the entry of cells into mitosis depends upon inhibition of synthetic processes involving phosphorylation. It is interesting that from the non-protein moiety of a phosphorylase has been isolated a yellow pigment tentatively identified as 2-methyl-1:4-naphthoquinone (Buell, 1952). It seems unlikely that at the concentrations used there is interference with glycolysis (Gemmill, 1949).

The comparison of the cytological effects of different compounds of closely related chemical constitution and also of ionising radiations makes it possible to separate some of the main types of mechanism of action of these agents on proliferating cells as follows :

(*a*) Blockage of the entry of cells into mitosis, without specification of the exact time of the action and presumably including pre-prophase and antephase inhibition. This is the characteristic action of Compound I, and in this case is associated with relatively few chromosomal abnormalities. Other less clearly defined examples are Compounds X and XV.

(*b*) Metaphase arrest and disturbances of the spindle mechanism apparently similar to the effects of colchicine. Examples of this type of action in association with (*a*) are Compounds II, VI, VIII, XI, XXVII and XXX at the higher concentrations, Compound IX at low concentrations and Compound XXVIII over a wide range of concentrations. Iso-naphthazarin produces metaphase arrest with little or no inhibition of the entry of cells into mitosis.

(*c*) Chromosome, including chromatid, breakage with varying degrees of rejoinability of the broken ends. Chromosome breakage without anaphase bridge formation is found with Compounds VIII, IX, XII, XIII, XIV and XXX. Chromosome breakage with anaphase bridge formation is observed characteristically with X-radiation and with Compound XXVIII ; considerably fewer anaphase bridges are observed with Compound XI. In all these instances chromosome breakage is associated with blockage of the entry of cells into mitosis. With Vitamin $K_1$ (Compound XXIX) there is no mitotic inhibition, but chromosome breakage and anaphase bridges are observed together with some spindle abnormalities.

In association with chromosome breakage, chromosomes showing regions of impaired stainability often with irregular and beaded structure are found with Compounds I (in low frequency), IX, XI and XXVIII ; similar changes can occur but are infrequent with X and $\gamma$ rays. These appearances suggest the possibility of two mechanisms of chromosome breakage, namely, "direct action" breaks similar to those produced by the passage of an ionising particle through or near a chromosome thread, and "metabolic" breaks due to impaired synthesis of the molecular structure of the chromosome.

It seems possible to assess very roughly the toxicity of a compound by observing the effects on the resting cells in the chick fibroblast cultures. Characteristic appearances are that the resting cells become disconnected and rounded up, with retraction of the cell processes. Changes of this type are seen with Compounds VIII, XI, XIV, XIX, XXV, XXVI and XXX in concentration $1 \times 10^{-5}$M or less. Our own animal experiments confirmed the high toxicity of Compound VIII.

The evidence obtained in these cytological studies suggests that the tetrasodium salt of 2:3-dimethyl-1:4-naphthohydroquinone diphosphate, Compound

XXVIII, is the most suitable compound for further investigation as a possible therapeutic radiosensitiser. It has been confirmed experimentally in the chick fibroblast cultures (Table III) that the combination of the effects of Compound XXVIII and of small doses of X-radiation shows potentiation of mitotic inhibition. The mitotic inhibition observed with the combined agents was so great that it was not possible to make satisfactory quantitative studies of chromosome fragmentation. It is of interest that in animal experiments Compound XXVIII is of low toxicity, and accumulates in the actively growing parts of the Walker Rat Carcinoma 256 and some other tissues after intramuscular injection, as shown by the fluorescence method used previously for Compound I (Mitchell, 1950, 1951). Clinical trials have been started.

Another compound which appears to merit further examination is the tetra-sodium salt of 2-methyl-3 bromo-1:4-naphthohydroquinone diphosphate, Compound IX.

SUMMARY.

1. The cytological effects of 29 selected compounds related to tetra-sodium 2-methyl-1:4-naphthohydroquinone diphosphate have been examined from the points of view of the relation of the biological actions to the chemical constitution and the selection of new compounds as possible therapeutic radiosensitisers.

2. The comparison of the cytological effects of the closely related compounds and also of ionising radiations makes it possible to separate three main types of mechanism of action on proliferating cells :

(a) blockage of the entry of cells into mitosis,
(b) metaphase arrest and spindle abnormalities apparently similar to the effects of colchicine, and
(c) chromosome breakage and its effects.

The appearances suggest the possibility of two types of chromosome breaks, which may be termed " direct action " breaks and " metabolic " breaks.

3. These biological effects are not directly dependent upon interactions with substances containing sulphydryl groups, although it is likely that this type of interaction plays some part.

4. It is suggested that inhibition of the entry of cells into mitosis depends on blockage of cellular synthetic processes involving phosphorylation.

5. These experiments suggest that the tetra-sodium salt of 2:3-dimethyl-1:4-naphthohydroquinone diphosphate, Compound XXVIII, is the most suitable compound for further investigation as a possible therapeutic radiosensitiser.

In addition to the help acknowledged in the text, Dr. E. J. Friedmann has given permission to refer to some of his unpublished work, especially that on phenols. We are grateful to Dr. Malcolm Dixon for suggesting the desirability of examining the dimethyl derivative, Compound XXVIII. We wish to thank Miss D. Brown and Mr. E. W. Mitchell for skilled technical assistance.

REFERENCES.

Barber, H. N., and Callan, H. G.—(1943) *Proc. Roy. Soc.*, B, **131**, 258.
Barron, E. S. G., and Dickman, S.—(1949) *J. gen. Physiol.*, **32**, 595.
Bourne, M. C.—(1937) *Physiol. Rev.*, **17**, 1.
Brachet, J.—(1947) 'Embryologie Chimique.' Paris (Masson).

BRAGG, L., KENDREW, J. C., AND PERUTZ, M. F.—(1950) *Proc. Roy. Soc.*, A, **203**, 321.
BUELL, M. V.—(1952) *Fed. Proc.*, **11**, 192.
CLOWES, G. H. A.—(1951) *Ann. N.Y. Acad.*, *Sci.*, **51**, 1409.
DAM, H.—(1942) 'Advances in Enzymology.' Edited by Nord, F. F., and Werkman, C. H. New York (Interscience), vol. 2, p. 286.
*Idem*, GLAVIND, J., AND KARRER, P.—(1940) *Helv. chim. acta.*, **23**, 224.
FIESER, L. F.—(1941) 'Experiments in Organic Chemistry.' Boston (D. C. Heath & Co.).
*Idem* AND FIESER, M.—(1944) 'Organic Chemistry.' Boston (D. C. Heath & Co.).
FRIEDMANN, E.—(1952) *Biochim. biophys. Acta* (in press).
*Idem* AND BAILEY, N. T. J.—(1950) *Ibid.*, **6**, 274.
*Idem*, MARRIAN, D. H., AND SIMON-REUSS, I.—(1948*a*), *Brit. J. Pharmacol.*, **3**, 263.—(1948*b*) *Ibid.*, **3**, 335.—(1949) *Ibid.*, **4**, 105.—(1952*a*), *Biochim. biophys. Acta* (in press).—(1952*b*) *Ibid.* (in press).
*Idem* AND SIMON-REUSS, I.—(1951) *Experientia*, **7**, 342.
GEMMILL, C. L.—(1949) *J. Pharmacol.*, **95**, 166.
HARTLEY, G. S., AND ROBINSON, C.—(1952) *Trans. Faraday Soc.* (in press).
KITCHEN, R., AND SANDIN, R. B.—(1945) *J. Amer. chem. Soc.*, **67**, 1645.
LEVINE, M.—(1951) *Ann. N.Y. Acad. Sci.*, **51**, 1365.
LOOMIS, W. F., AND LIPMANN, F.—(1948) *J. biol. Chem.*, **173**, 807.
MCELROY, W. D.—(1947) *Quart. Rev. Biol.*, **22**, 25.
MARRIAN, D. H.—(1949*a*) *J. chem. Soc.*, 1515.—(1949*b*), *Ibid.*, 1797.
MITCHELL, J. S.—(1949) *Ann. Rep. Brit. Emp. Cancer Campgn.*, **27**, 214.—(1950) *Ibid.*, **28**, 213.—(1951) *Ibid.*, **29** (in press).
*Idem* AND SIMON-REUSS, I.—(1952) *Brit. J. Cancer*, **6**, 305.
PATT, H. M., SMITH, D. E., TYREE, E. B., AND STRAUBE, R. L.—(1950) *Proc. Soc. exp. Biol.*, *N.Y.*, **73**, 18.
TARBELL, D. S., FUKUSHIMA, D. K., AND DAM, H.—(1945) *J. Amer. chem. Soc.*, **67**, 1643.
TRENNER, N. R., AND BACHER, F. A.—(1941) *J. biol. Chem.*, **137**, 745.
VILLEE, C. A., LOWENS, M., GORDON, M., LEONARD, E., AND RICH, A.—(1949) *J. cell. comp. Physiol.*, **33**, 93.
WILLIAMS, R. T.—(1947) 'Detoxication Mechanisms.' London (Chapman & Hall, Ltd.).